# Intrinsic PD‐L1 Degradation Induced by a Novel Self‐Assembling Hexapeptide for Enhanced Cancer Immunotherapy

**DOI:** 10.1002/advs.202410145

**Published:** 2024-11-12

**Authors:** Hongxia Zhang, Ming Ji, Yamei Wang, Mengmeng Jiang, Zongyu Lv, Gongyu Li, Lulu Wang, Zhen Zheng

**Affiliations:** ^1^ The Province and Ministry Co‐sponsored Collaborative Innovation Center for Medical Epigenetics Tianjin Key Laboratory on Technologies Enabling Development of Clinical Therapeutics and Diagnostics School of Pharmacy Tianjin Medical University Tianjin 300070 China; ^2^ Tianjin Key Laboratory of Biosensing and Molecular Recognition Research Center for Analytical Science Frontiers Science Center for New Organic Matter College of Chemistry Nankai University Tianjin 300071 China

**Keywords:** carrier‐free oncotherapy, PD‐L1 downregulator, self‐assembly peptide, supramolecular hydrogel

## Abstract

Programmed death‐ligand 1 (PD‐L1) is a critical immune checkpoint protein that facilitates tumor immune evasion. While antibody‐based PD‐1/PD‐L1 inhibitors have shown promise, their limitations necessitate the development of alternative therapeutic strategies. This work addresses these challenges by developing a hexapeptide, **KFM** (Lys‐Phe‐Met‐Phe‐Met‐Lys), capable of both directly downregulating PD‐L1 and self‐assembling into a ROS‐responsive supramolecular hydrogel. This dual functionality allows **Gel KFM** to function as a localized drug delivery system and a PD‐L1 inhibitor. Loading the hydrogel with mitoxantrone (MTX) and metformin (MET) further enhances the therapeutic effect by combining chemotherapy with PD‐L1 downregulation. In vitro and in vivo studies demonstrate significant tumor growth inhibition, increased CD8+ T cell infiltration, and reduced intratumoral PD‐L1 expression following peritumoral administration. Mechanistically, **KFM** promotes PD‐L1 degradation via a ubiquitin‐dependent pathway. This “carrier‐free” delivery system expands the role of supramolecular hydrogels beyond passive carriers to active immunotherapeutic agents, offering a promising new strategy for cancer therapy.

## Graphical abstract

This paper reports a novel hexapeptide system that promotes PD‐L1 degradation through ubiquitin‐dependent pathways while forming ROS‐responsive supramolecular hydrogels. This dual‐function, carrier‐free system shows significant antitumor efficacy and immune activation (in vitro and in vivo) when combined with chemotherapeutics, transforming hydrogels from passive carriers into active immunotherapeutic agents and establishing a novel platform for cancer immunotherapy.

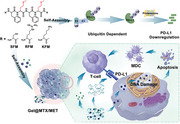

## 1 Introduction

Programmed death‐ligand 1 (PD‐L1), a critical immune checkpoint protein, is considered as the major mechanism for immune invasion of cancer cells.^[^ ^1^ ^]^ The PD‐1/PD‐L1 interaction between tumor cells and T‐cells inhibits cytotoxic T‐cell activity, enabling cancer cells to escape immune surveillance.^[^ ^2^ ^]^ Immunotherapy, especially antibody‐based PD‐1/PD‐L1 inhibitors, such as Pembrolizumab and Nivolumab, has yielded sustained antitumor responses and prolonged patient survival in the clinic.^[^ ^3^ ^]^ However, therapeutic blocking antibodies targeting PD‐1/PD‐L1 face challenges, including immune‐related adverse events,^[^ ^4^ ^]^ potential antigenic reactions, high economic costs, and limited tumor tissue permeability due to large molecular size and stability.^[^ ^5^ ^]^ These issues necessitate the exploration of alternative nonantibody PD‐L1 therapeutics and innovative delivery systems to elevate clinical benefits and alleviate economic burdens.^[^ ^6^ ^]^

Downregulating PD‐L1 expression, rather than solely blocking its interaction with PD‐1, may be a promising strategy for a durable antitumor immune response.^[^ ^7^ ^]^ By reducing PD‐L1 accumulation in the tumor cells, these agents can potentiate the activity of tumor‐infiltrating T cells and potentially enhance long‐term antitumor immunity.^[^ ^8^ ^]^ Several small‐molecule transcriptional/epigenetic modulators toward PD‐L1 are currently in early clinical trials.^[^ ^9^ ^]^ However, the limited availability of potent and selective PD‐L1 downregulators highlights the urgent need for further optimization of these compounds to meet the rigorous regulatory requirements for clinical translation.^[^ ^10^ ^]^ Peptides, chains of amino acids linked by peptide bonds, represent a hybrid class of therapeutics with synthetic simplicity, biological compatibility, and remarkable versatility.^[^ ^11^ ^]^ Despite their theoretical advantages, only a limited number of PD‐L1 targeting/binding peptides have been reported, and no efficient PD‐L1 downregulating peptide has been discovered thus far.^[^ ^12^ ^]^

Additionally, researchers have begun exploring alternative therapeutic platforms that could effectively deliver PD‐L1 downregulators to the tumor site.^[^ ^13^ ^]^ In this regard, supramolecular hydrogels have emerged as a focal point for localized cancer therapy, particularly for eradicating residual tumor cells post‐surgery to prevent recurrence.^[^ ^14^ ^]^ Localized delivery via supramolecular hydrogels confers enhanced drug stability, sustained drug presence, and minimized systemic toxicity compared to systemic intravenous injection,^[^ ^15^ ^]^ which is frequently associated with considerable toxicity, adverse effects, and inefficient drug utilization.^[^ ^16^ ^]^ Furthermore, supramolecular hydrogels as platforms offer versatility in combining with other therapeutic agents, that synergize the effects of chemotherapy, immunotherapy, and gene therapy, potentially amplifying the antitumor efficacy.^[^ ^17^ ^]^ To date, the utilization of supramolecular hydrogels in oncology has been largely restricted as carriers for controlled drug delivery.^[^ ^18^ ^]^ Hence, the discovery of peptides capable of both self‐assembling into supramolecular hydrogels and simultaneously downregulating PD‐L1 expression presents a compelling advancement in cancer therapy, demonstrating their active role in modulating the tumor immune microenvironment beyond merely facilitating drug release.

**Scheme 1:**
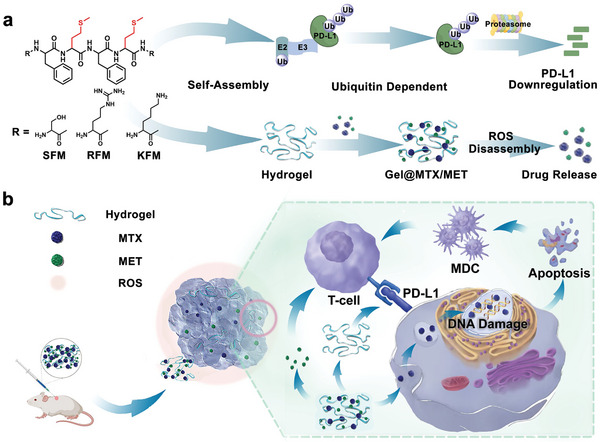
a) Chemical structures of the hexapeptides and their mechanism for PD‐L1 downregulation. b) Application of the self‐assembling hexapeptides in forming ROS‐responsive supramolecular hydrogels for carrier‐free cancer immunotherapy. MDC, Mature Dendritic Cells.

Inspired by the promising applications of self‐assembling peptides in cancer therapy, herein, we rationally designed a hexapeptide, Lys‐Phe‐Met‐Phe‐Met‐Lys (**KFM**), which incorporates specific amino acids to confer distinct physicochemical properties crucial for its function in PD‐L1 downregulation and localized delivery. Lysine residues were strategically positioned at both termini of the peptide to provide a positive charge to disrupt the electrostatic interactions that stabilize the cytoplasmic domain of PD‐L1 (PD‐L1‐CD), leading to its membrane dissociation and subsequent degradation.^[^ ^19^ ^]^ The inclusion of phenylalanine residues was motivated by their hydrophobic nature, which promotes π–π stacking interactions. These interactions are critical for the peptide's self‐assembly into a hydrogel structure, enabling localized and sustained delivery at the tumor site. Methionine residues were incorporated to confer responsiveness to reactive oxygen species (ROS), which are often present at elevated levels in the tumor microenvironment.^[^ ^20^ ^]^ Utilizing the FDA‐approved DNA topoisomerase II poison Mitoxantrone (MTX) to induce DNA damage and cytotoxicity,^[^ ^21^ ^]^ and Metformin (MET) to further enhance CD8+ T cell activity,^[^ ^22^ ^]^ this study showcases the formulation of encapsulation of MTX and MET within the PD‐L1 downregulating hydrogel, followed by peritumoral administration for carrier‐free oncotherapy (**Scheme** 1). This dual‐action hydrogel serves a multifaceted role in cancer therapy. The incorporation of chemo‐drug within the hydrogel matrix contributes to reduced tumor burden and an enhanced antitumor immune microenvironment.^[^ ^23^ ^]^ Concurrently, the suppression of PD‐L1 by the hydrogel itself unleashes the anti‐tumor immune response, augmenting the therapeutic efficacy against tumor progression and metastasis. Both in vitro and in vivo studies highlight the potential of the PD‐L1 modulating hydrogel as a promising platform for targeted drug delivery and combination therapy. Mechanistic studies revealed that treatment with these peptides and their subsequent hydrogel formation induces ubiquitination‐dependent proteasomal degradation of PD‐L1. This research introduces a novel, dual‐action, hexapeptide‐based hydrogel that functions as both a “carrier‐free” delivery system and a potent PD‐L1 downregulator. Notably, it positions supramolecular hydrogels as active agents in the elimination of PD‐L1 in the context of cancer therapy.

## 2 Results and Discussion

### 2.1 Hexapeptide Induced PD‐L1 Downregulation

**Figure 1:**
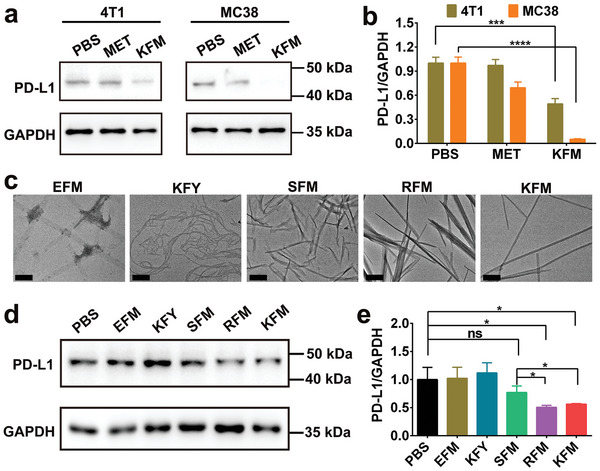
Peptide‐induced PD‐L1 downregulation. a) Western blot analysis of PD‐L1 expression in 4T1 and MC38 cells after treatment with MET and **KFM** peptide, respectively. b) Quantitative data of PD‐L1 expression levels in 4T1 and MC38 cells. c) Representative TEM images of self‐assembled peptides **EFM**, **KFY**, **SFM**, **RFM,** and **KFM**. Scale bar: 500 nm. d) Western blot analysis of PD‐L1 expression in MC38 cells treated with various peptides. e) Quantitative data of PD‐L1 expression levels in MC38 cells following peptide treatments. Statistical significance is denoted as ^*^ *p* < 0.05, ^***^ *p* < 0.001, and ^****^ *p* < 0.0001.

While peptides have shown potential as immunologic adjuvants capable of inducing systemic immune responses, their influence on immune checkpoint molecules has not been reported. In this study, we first synthesized the hexapeptide **KFMFMK** (Lys‐Phe‐Met‐Phe‐Met‐Lys, **KFM**) using solid‐phase peptide synthesis (SPPS). The synthesized peptide was then purified by high‐performance liquid chromatography (HPLC) and verified by high‐resolution mass spectrometry (HRMS) (Figure S1, Supporting Information). Recognizing the critical role of PD‐L1 in tumor immune evasion, we first investigated the impact of **KFM** on PD‐L1 expression in tumor cells. MET, previously reported to induce PD‐L1 degradation,^[^ ^24^ ^]^ served as a positive control. Western blot analysis revealed that both MET and **KFM** reduced PD‐L1 expression in both 4T1 and MC38 cell lines (**Figure** **1**a,b). Notably, **KFM** exhibited significantly greater potency in suppressing PD‐L1 expression compared to MET, achieving relative PD‐L1 levels of 0.491 and 0.052 in 4T1 and MC38 cells, respectively, compared to untreated controls. To further elucidate the mechanism of action, we conducted additional Western blot analyses comparing the PD‐L1 downregulation capabilities of **KFM** in its self‐assembled form (hydrogel) versus the freshly prepared **KFM** peptide monomers in their nonassembled state. Our results (Figure S2, Supporting Information) revealed a striking difference: only the self‐assembled **KFM** hydrogel demonstrated effective PD‐L1 downregulation, while the nonassembled **KFM** monomers showed a negligible effect. This finding underscores the critical role of self‐assembly and subsequent hydrogel formation in conferring the therapeutic efficacy of our system. The self‐assembled nanostructures likely provide a unique spatial arrangement or increased local concentration of the active peptide, enabling more efficient downregulation of PD‐L1.

To explore the generalizability of hexapeptides in inhibiting PD‐L1 expression, we synthesized a series of hexapeptides with varying charge and amino acid composition, including negatively charged **EFMFME** (Glu‐Phe‐Met‐Phe‐Met‐Glu, **EFM**), neutral **SFMFMS** (Ser‐Phe‐Met‐Phe‐Met‐Ser, **SFM**), positively charged **RFMFMR** (Arg‐Phe‐Met‐Phe‐Met‐Arg, **RFM**), and **KFYFYK** (Lys‐Phe‐Tyr‐Phe‐Tyr‐Lys, **KFY**), which lacks methionine. Transmission electron microscopy (TEM) images confirmed the self‐assembly ability of these peptides, revealing the formation of diverse fibrous nanostructures (Figure 1c). Consistent with the initial findings on **KFM**, treatment with **SFM**, **RFM**, and **KFM** resulted in varying degrees of PD‐L1 downregulation in MC38 cells compared to the PBS control group (Figure 1d,e). Significant suppression of PD‐L1 expression was observed with both **RFM** and **KFM** treatments, suggesting the importance of both methionine and positive charge in mediating this effect. Based on its superior ability to downregulate PD‐L1 and its favorable hydrogel‐forming properties, **KFM** was selected as the carrier for subsequent oncotherapy studies.

### 2.2 Characterization of Gel KFM and H_2_O_2_‐Instructed Disassembly

**Figure 2:**
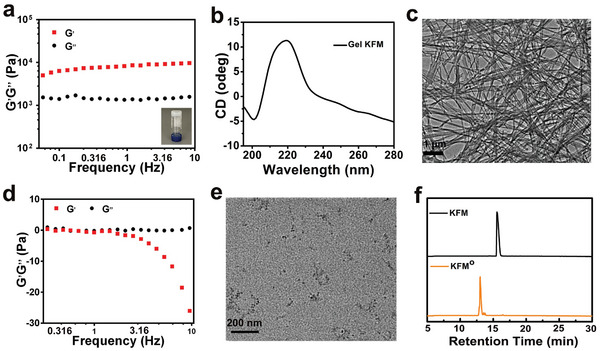
Characterization of **Gel KFM** and H_2_O_2_‐stimulated disassembly. a) Frequency sweep rheological analysis of the **Gel KFM** (Insets: images of corresponding hydrogels). b) CD spectrum of **Gel KFM**. c) Representative TEM image of **Gel KFM**. d) The frequency sweep (0.1–10 Hz) rheological analysis of **Gel KFM** (0.5 wt.%) after incubation with 10 mm H_2_O_2_ overnight (strain: 0.1%). e) TEM image of **Gel KFM** after incubation with 10 mm H_2_O_2_ overnight. f) HPLC traces of **Gel KFM** after incubation with cell lysates overnight. Detection wavelength: 254 nm.

Having identified **KFM** as a potent suppressor of PD‐L1 expression with promising hydrogel‐forming capabilities, we next sought to characterize the physicochemical properties of the resulting hydrogel, **Gel KFM**. Rheological analysis revealed that the storage modulus (G') of **Gel KFM** was significantly higher than the loss modulus (G″) across the tested frequency (0.1–10 Hz) and strain (0.01–10%) ranges, indicating the formation of a stable gel capable of withstanding external shear forces (**Figure** **2**a; Figure S3a, Supporting Information). Circular dichroism (CD) spectroscopy was employed to elucidate the secondary structure within the hydrogels. The CD spectra of **Gel KFM** exhibited negative peaks ≈200 nm and positive peaks ≈220 nm, indicative of a β‐sheet structure (Figure 2b). TEM images of **Gel KFM** (Figure 2c) revealed densely packed nanofibers with average diameters of 53 ± 1.8 nm. Importantly, these structural properties of **Gel KFM** remained unaffected by drug incorporation, with drug‐loaded hydrogels exhibiting similar rheological properties, CD patterns, nanofiber morphology, and average diameters (47 ± 2.3 nm) (Figure S4, Supporting Information). This observation underscores the potential of **Gel KFM** as a favorable drug delivery vehicle. Details regarding the preparation and characterization of the drug‐loaded hydrogel, **Gel@MTX/MET**, are provided in the Supporting Information.

Additionally, the presence of methionine in **KFM**, which is susceptible to oxidation by hydrogen peroxide (H_2_O_2_), endows **Gel KFM** with H_2_O_2_‐responsive properties. This sensitivity to H_2_O_2_, abundant in the tumor microenvironment,^[^ ^25^ ^]^ enables hydrogel disassembly at the tumor site. As expected, incubation with H_2_O_2_ overnight induced a gel‐to‐sol transition in **Gel KFM**, while PBS‐treated hydrogels remained in their gel state. This transition was further confirmed by alterations in rheological properties (Figure 2d; Figure S3b, Supporting Information) and TEM imaging (Figure 2e), which revealed a shift from the initial nanofiber structure of **Gel KFM** to an amorphous morphology following H_2_O_2_ incubation. To verify the chemical basis of this gel‐to‐sol conversion, we performed HPLC and MS analyses. The HPLC profile and MS results (Figure 2f; Figure S5, Supporting Information) clearly demonstrate that incubation with H_2_O_2_ converted the hydrogelator **KFM** to its hydrophilic form, **KFM°**. This conversion is attributed to the oxidation of methionine residues to methionine sulfoxide in **KFM°**, disrupting the hydrophobic interactions essential for hydrogel integrity and leading to disassembly. Intriguingly, WB analysis revealed that **KFM°** lost the ability to degrade PD‐L1 (Figure S2, Supporting Information). This loss of function could be attributed to either the oxidation of methionine or a disruption in peptide self‐assembly, both of which are critical to its mechanism of action.

The drug‐loaded hydrogel, **Gel@MTX/MET**, was prepared as described in the Supplementary Information. The average encapsulation efficiencies were determined to be 95.8% ± 2.2% for MTX and 98.5% ± 3.5% for MET, confirming the successful encapsulation of both drugs within the hydrogel matrix. Similarly, **Gel@MTX/MET** underwent a gel‐to‐sol transition upon overnight incubation with H_2_O_2_, while PBS‐treated hydrogels remained intact (Figure S6, Supporting Information). Rheological analysis further confirmed this H_2_O_2_‐triggered transformation (Figure S7a,b, Supporting Information). To assess the drug release kinetics from **Gel KFM**, we monitored drug release over a five‐day period. In the presence of 100 µm H_2_O_2_, the release rates of MET and MTX from **Gel KFM** reached 89.73% and 86.79%, respectively, after 120 h. In contrast, only 25.11% of MET and 20.67% of MTX were released in the absence of H_2_O_2_ (Figure S7c,d, Supporting Information). These findings demonstrate that **Gel KFM** possesses both ROS‐responsiveness and controlled drug release capabilities.

### 2.3 In Vitro Cytotoxicity and Immunogenic Cell Death Effect

**Figure 3:**
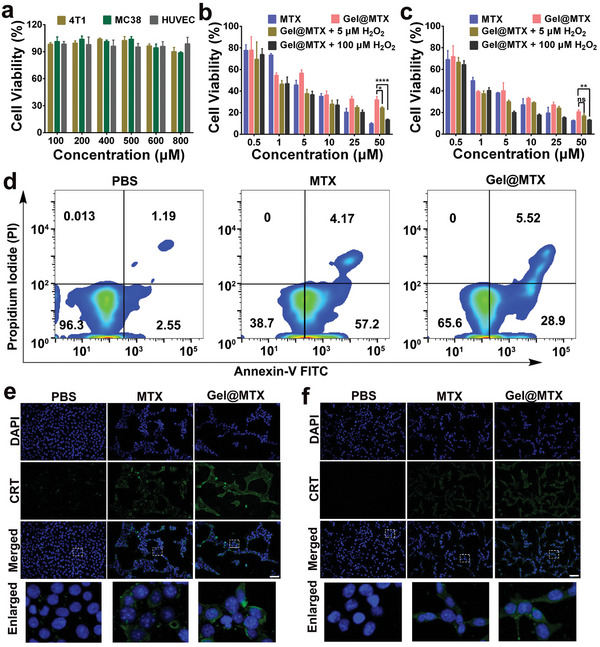
Cytotoxicity assays. a) MTT assay of **KFM** peptide against 4T1, MC38, and HUVEC cells. Cytotoxicity of b) 4T1 or c) MC38 tumor cells with different treatments. d) Flow cytometry analysis of apoptosis in 4T1 cells following 24‐h exposure to MTX (5 µm) and **Gel@MTX** (equivalent to 5 µm MTX) in the absence of H_2_O_2_. Fluorescence images of CRT staining in e) 4T1 tumor cells and f) MC38 tumor cells following treatment with PBS, MTX, and **Gel@MTX**. Scale bar: 100 µm. The white dashed rectangle indicates the zoomed‐in region.

To assess the safety and efficacy of the developed drug delivery system, we evaluated the biocompatibility of the hydrogelator **KFM** and its oxidized counterpart **KFM°** against 4T1, MC38, and HUVEC cells. As shown in **Figure** **3**a and Figure S8 (Supporting Information), **KFM** and **KFM°** both exhibited negligible cytotoxicity across all three cell lines, indicating their favorable biocompatibility. Next, we assessed the cytotoxicity of our drug‐loaded system under various conditions. As shown in Figure S9 (Supporting Information), H₂O₂ concentrations below 100 µm exhibited negligible cytotoxicity on 4T1 and MC38 cells, allowing us to subsequently investigate the combined effects of H₂O₂ and MTX formulations without significant interference from H₂O₂ itself. We compared the effects of free MTX, **Gel@MTX**, and **Gel@MTX** in the presence of H_2_O_2_ (to mimic the oxidative tumor microenvironment) on both 4T1 and MC38 cell lines. Free MTX demonstrated dose‐dependent cytotoxicity, while encapsulation of MTX within the hydrogel resulted in reduced cytotoxicity, suggesting a sustained release profile of MTX from the hydrogel matrix (Figure 3b,c). The addition of a low concentration of H_2_O_2_ (5 µM) to **Gel@MTX** resulted in a modest enhancement of its cytotoxic effect compared to **Gel@MTX** alone. However, a more pronounced decrease in cell viability was observed as the H_2_O_2_ concentration increased to 100 µm. This enhanced efficacy can be attributed to the H_2_O_2_‐mediated acceleration of MTX release from the hydrogel, as demonstrated in our release kinetics study (Figure S7d, Supporting Information). The accelerated release leads to higher local concentrations of MTX, thereby augmenting its cytotoxic effects on cancer cells. Given the apoptosis‐inducing property of MTX, we further investigated the apoptotic potential of **Gel@MTX** (equivalent to 5 µm MTX) in the absence of H_2_O_2_ using flow cytometry. As shown in Figure 3d, the MTX (5 µm ) group exhibited the highest percentage of apoptotic cells (61.3%), which was greater than that observed in the **Gel@MTX** treatment group (34.4%). This difference can be attributed to the encapsulation of MTX within the **KFM** hydrogel, which modulates its release and consequently reduces immediate cytotoxicity. Similar results were observed in MC38 cells (Figure S10, Supporting Information). Having verified the cell death mechanism for **Gel@MTX**‐induced apoptosis, we next examined the downstream effects of this process. Chemotherapeutic agents like MTX have been reported to induce immunogenic cell death (ICD),^[^ ^26^ ^]^ a critical process for eliciting robust systemic antitumor immunity and enhancing the efficacy of PD‐1/PD‐L1 blockade.^[^ ^27^ ^]^ ICD is characterized by the release of damage‐associated molecular patterns (DAMPs),^[^ ^28^ ^]^ such as calreticulin (CRT) and high mobility group box 1 protein (HMGB1), which activate potent innate immune responses.^[^ ^29^ ^]^ To evaluate the ability of our system to induce ICD, we assessed CRT and HMGB1 expression in treated 4T1 and MC38 cells. Treatment with both free MTX and **Gel@MTX** led to a marked increase in CRT expression, indicated by elevated fluorescence signal, compared to the PBS control (Figure 3e). Notably, **Gel@MTX** exhibited the strongest fluorescence intensity, suggesting enhanced ICD induction by the MTX‐**Gel KFM** combination, with a 3.04‐fold increase compared to MTX alone and an 18.16‐fold increase compared to the PBS control (Figure S11, Supporting Information). Consistent with these findings, **Gel@MTX** treatment also resulted in detectable green fluorescence of HMGB1, indicating its translocation from the nucleus to the extracellular matrix (Figure S12, Supporting Information). Similar results were observed in **Gel@MTX**‐treated MC38 cells (Figure 3f; Figures S13 and S14, Supporting Information). Taken together, these results demonstrate that the MTX‐**Gel KFM** combination effectively induces ICD in both 4T1 and MC38 cells. This ICD induction has the potential to enhance antigen presentation,^[^ ^30^ ^]^ thereby promoting a more robust antitumor immune response and increasing sensitivity to PD‐1/PD‐L1 blockade.^[^ ^31^ ^]^

### 2.4 In Vivo Antitumor Study on 4T1 and MC38 Tumor‐Bearing Mice

We first conducted a retention study to assess the in vivo metabolism and degradation profile of the hydrogel. As shown in Figure S15 (Supporting Information), free MTX was barely detectable 12 h postinjection in mice. In contrast, the **Gel@MTX** treatment group still showed a small amount of MTX present at 60 h postinjection. Importantly, **Gel@MTX** was completely metabolized by 72 h after administration. These results demonstrate that our biodegradable peptide‐based hydrogel significantly prolongs the retention time of the encapsulated drug while avoiding potential systemic toxicity associated with long‐term retention.

To evaluate the in vivo antitumor efficacy of our PD‐L1‐modulating hydrogel, we employed preclinical 4T1 and MC38 tumor‐bearing mouse models. Prior to large‐scale studies, we confirmed the PD‐L1 downregulating ability of **Gel KFM** in vivo using western blot analysis. As shown in Figure S16 (Supporting Information), preliminary results demonstrated the hydrogel's capacity to effectively downregulate PD‐L1 expression in vivo.

**Figure 4:**
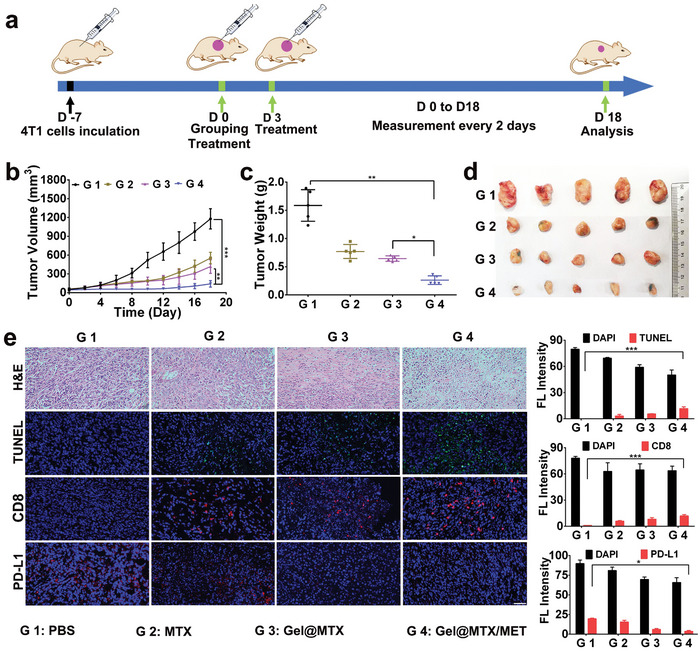
Therapeutic effects of **Gel@MTX/MET** in vivo. a) Schematic iconography of the therapeutic protocol of the mice bearing 4T1 tumors. b) Tumor growth curve for mice intratumorally injected with PBS, MTX, **Gel@MTX**, and **Gel@MTX/MET**. c) The endpoint of tumor weight of 4T1‐tumor bearing mice. d) Representative tumor images of all the mice at 18 days post‐treatment. e) Immunofluorescence images showing H&E, TUNEL, CD8, and PD‐L1 staining in the tumor region of mice at 18 days post‐treatment. Following the quantitative data of the immunofluorescence fluorescence intensity of TUNEL, CD8, and PD‐L1. Scale bar: 100 µm. ^*^ *p <* 0.05, ^**^ *p <* 0.01, ^***^ *p <* 0.001.

In the 4T1 tumor model, mice with an average tumor volume of 60 mm^3^ were randomly assigned to four treatment groups: PBS, MTX, **Gel@MTX**, and **Gel@MTX/MET** (**Figure** **4**a). The PBS‐treated group exhibited rapid tumor growth, with volumes reaching ≈1200 mm^3^ by 18 days postadministration. In contrast, all drug‐treated groups showed significant tumor growth suppression. Notably, mice treated with **Gel@MTX** or **Gel@MTX/MET** exhibited stronger tumor growth inhibition compared to those treated with free MTX (Figure 4b–d). This enhanced efficacy is attributed to the synergistic effect of controlled MTX release facilitated by gel encapsulation and the PD‐L1 downregulation mediated by **Gel KFM**. Histological analysis further confirmed these antitumor effects, revealing the highest levels of necrotic lesions and apoptosis rates in the **Gel@MTX/MET**‐treated group (Figure 4e).

Building on these encouraging histological findings, we sought to elucidate the underlying immunological mechanisms driving the enhanced therapeutic efficacy of **Gel@MTX/MET**. We monitored the immune activation at 3‐day intervals following each dose administration. As illustrated in Figures S17 and S18 (Supporting Information), the **Gel@MTX/MET** treatment induced a pronounced enhancement in CD8+ T cell infiltration, with nearly threefold and fourfold increases observed at days 3 and 6, respectively, compared to the PBS control group. Conversely, PD‐L1 expression in the **Gel@MTX/MET** group decreased by approximately twofold relative to the PBS group on both days 3 and 6. Notably, this favorable immune activation persisted until the experimental endpoint (day 18). At this time point, the **Gel@MTX/MET** treatment group exhibited significantly higher infiltration of CD8+ T cells (10.94%) and a markedly lower proportion of PD‐L1‐positive tumor cells (3.70%) compared to other treatment groups (Figure 4e). These findings support the establishment of an antitumor immune microenvironment and effective immune checkpoint inhibition.

**Figure 5:**
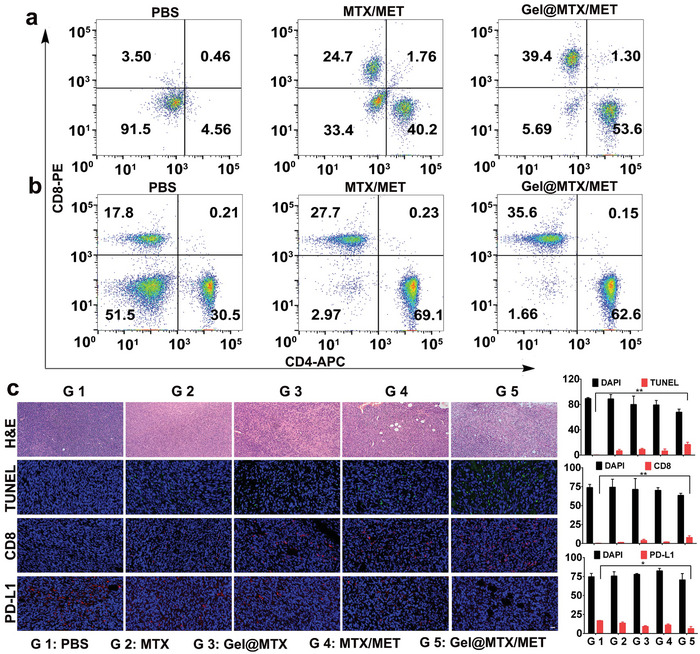
Immune activation induced by **Gel@MTX/MET** treatment in vivo. Flow cytometric analysis of CD4+ and CD8+ T cell populations in a) tumor tissue and b) spleens of tumor‐bearing mice following various treatments. c) Immunofluorescence images showing H&E, TUNEL, CD8, and PD‐L1 staining in the MC38 tumor region of mice at 18 days post‐treatment. Following the quantitative data of the immunofluorescence fluorescence intensity of TUNEL, CD8, and PD‐L1. Scale bar: 50 µm. ^*^ *p <* 0.05, ^**^ *p <* 0.01, ^***^ *p <* 0.001. G1: PBS, G2: MTX, G3: **Gel@MTX**, G4: **Gel@MTX/MET**.

Encouraged by the immunofluorescence staining results, we conducted a comprehensive flow cytometric analysis on tumor tissues from 4T1‐bearing mice. First, we evaluated dendritic cell (DC) maturation in tumor tissues. The **Gel@MTX/MET** group showed significantly increased proportions of CD80‐positive (7.01%) and CD86‐positive (6.53%) cells compared to the PBS group (Figure S19, Supporting Information). As mature DCs are crucial for activating naive T cells, we further analyzed CD4+ and CD8+ T cell activation in both tumor tissues and spleens. As shown in **Figure** **5**a,b, the MTX/MET and **Gel@MTX/MET** groups exhibited higher expression of CD4+ and CD8+ T cells compared to the PBS control. Notably, the **Gel@MTX/MET** group outperformed all other treatments, with CD4+ and CD8+ T cell populations increasing from 8.5% and 48.5% in the PBS group to 94.31% and 98.34%, respectively. These results collectively suggest that the **Gel@MTX/MET** formulation effectively induces DC maturation and robust T‐cell responses, contributing to its superior antitumor efficacy. The sustained immune activation and favorable modulation of the tumor microenvironment highlight the potential of this combinatorial approach in enhancing cancer immunotherapy outcomes.

Given the mild toxicity observed with the high dose of MTX (3 mg kg^−1^) in the 4T1 model, we further evaluated the antitumor efficacy of **Gel@MTX/MET** at a reduced dose in the MC38 tumor model. Subcutaneous MC38 tumor‐bearing mice received peritumoral injections of the different MTX formulations once the average tumor volume reached ≈60 mm^3^ (Figure S20a, Supporting Information). As shown in Figure S20b (Supporting Information), tumor growth in the PBS‐treated group continued unabated. While treatment with free MTX, **Gel@MTX**, or the combination of free MTX and MET partially slowed tumor growth, administration of the **Gel@MTX/MET** hydrogel led to significant tumor regression (Figure S20c,d, Supporting Information). Histological analysis of the MC38 tumors corroborated the therapeutic effect of **Gel@MTX/MET**, revealing the highest levels of tumor necrosis and apoptosis among all treatment groups (Figure 5c). Moreover, we detected robust CD8+ positive signals indicative of intra‐tumoral infiltrating lymphocytes in the **Gel@MTX/MET** group, further supporting the potent immune activation elicited by this treatment. Analysis of PD‐L1 expression in tumor tissues revealed a marked decrease in PD‐L1 expression in the **Gel@MTX/MET** group, with quantitative analysis demonstrating the lowest fluorescence intensity of PD‐L1 (6.58%) among all treatments, confirming efficient inhibition of PD‐L1 surface expression (Figure S21, Supporting Information).

Finally, we assessed the biocompatibility of our hydrogel‐based combination therapy in both preclinical mouse models by monitoring changes in body weight and conducting H&E staining of major organs. We observed no significant weight loss or any visible signs of inflammatory response or tissue injury, indicating the favorable biocompatibility of the **Gel@MTX/MET** formulation (Figures S22 and S23, Supporting Information). Overall, our findings demonstrate that **Gel@MTX/MET** effectively inhibits tumor growth in both 4T1 and MC38 syngeneic mouse models. This enhanced antitumor efficacy is mediated by a combination of controlled drug release, potent PD‐L1 downregulation, and robust activation of antitumor immunity. Importantly, **Gel@MTX/MET** exhibited an excellent safety profile in our preclinical studies, highlighting its potential as a promising therapeutic strategy for the treatment of cancers.

### 2.5 Mechanistic Study of Gel KFM Induced PD‐L1 Downregulation

**Figure 6:**
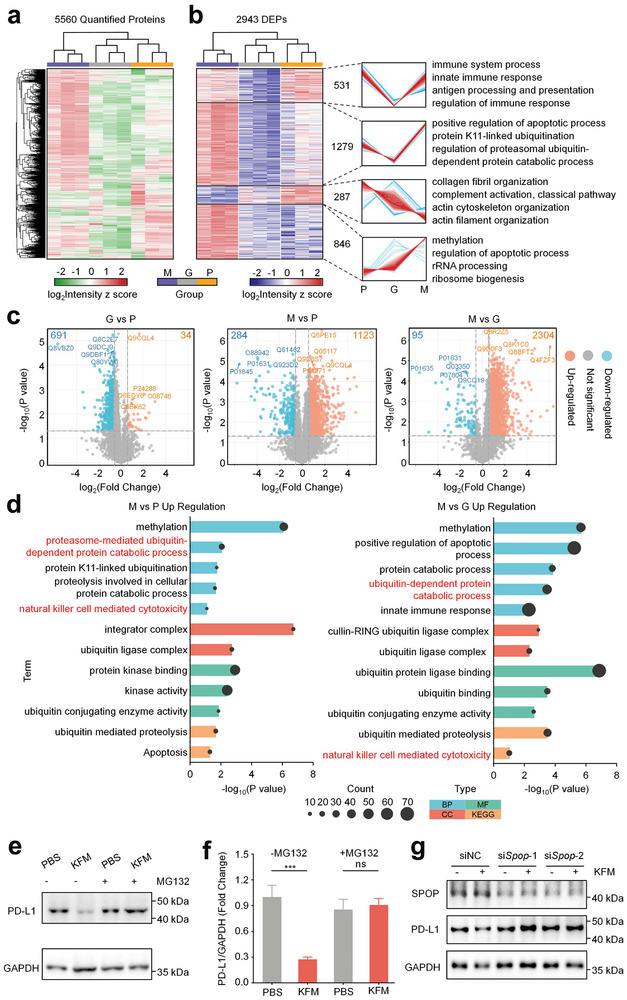
Proteomic profiling of tumor tissue across treatment groups. a) Hierarchical clustering of 5560 quantified proteins, showing distinct clustering of biological replicates in the PBS (P), **Gel KFM** (G), and **Gel@MTX/MET** (M) groups, indicating significant intergroup differences. b) Hierarchical clustering of 2943 significantly altered proteins, with the number of proteins and enriched biological processes/pathways for each cluster. c) Volcano plots of pairwise protein abundance comparisons, highlighting significantly differentially expressed proteins (*p* < 0.05, fold change > 1.5). Downregulated proteins are shown in blue, and upregulated in orange. d) GO and KEGG pathway analyses of the differentially expressed proteins. e) The Western Blot of PD‐L1 expression of 4T1 cells after treatments with or without MG132. f) Quantitative data of the PD‐L1 expression level of 4T1 cells. g) WB analysis of SPOP and PD‐L1 expression in MC38 cells following SPOP knockdown and **KFM** treatment. ^***^ *p* < 0.001.

To elucidate the mechanism underlying PD‐L1 suppression by our peptide hydrogels, we performed mass spectrometry (MS)‐based proteomic analysis of 4T1 tumor tissues treated with **Gel KFM**. Hierarchical clustering of quantified proteins revealed distinct proteomic profiles for each treatment group (PBS, **Gel KFM**, and **Gel@MTX/MET**), as evidenced by the clear separation of biological replicates within each group (**Figure** **6**a). Gene Set Variation Analysis (GSVA) further confirmed this robust clustering and identified significant intergroup differences in cellular components, biological processes, molecular functions, and pathways (Figure 6b–d). Notably, Gene Ontology (GO) enrichment and Kyoto Encyclopedia of Genes and Genomes (KEGG) pathway analyses highlighted the ubiquitin‐dependent protein catabolic process as a key pathway potentially implicated in hydrogel‐mediated PD‐L1 downregulation (Figure S24, Supporting Information). This pathway was characterized by the upregulation of genes involved in various aspects of ubiquitination, including those associated with the positive regulation of protein ubiquitination, ubiquitin‐conjugating enzyme activity, ubiquitin protein ligase binding, and ubiquitin binding.

To validate the role of the ubiquitin‐proteasome pathway in **Gel KFM**‐mediated PD‐L1 downregulation, we examined the effect of the proteasome inhibitor MG132 on **Gel KFM**‐treated cells. As shown in Figure 6e,f, MG132 treatment effectively attenuated the reduction in PD‐L1 protein levels induced by **Gel KFM**, confirming that this downregulation occurs at the protein level and is dependent on the proteasome. Interestingly, while **Gel KFM** treatment decreased PD‐L1 protein levels, we observed an increase in PD‐L1 mRNA expression (Figure S25, Supporting Information), suggesting a potential compensatory feedback mechanism in response to reduced PD‐L1 protein. Additionally, we investigated the role of the ubiquitin‐proteasome pathway, specifically focusing on the Speckle‐type POZ protein (SPOP). SPOP is a crucial substrate adaptor for the Cullin 3‐based E3 ligase (CRL3), which plays a significant role in protein ubiquitination and subsequent degradation.^[^ ^32^ ^]^ We conducted siRNA knockdown studies targeting SPOP to assess its involvement in the PD‐L1 regulatory pathway. As shown in Figure 6f, siRNA‐mediated knockdown of SPOP (siSpop) effectively reduced SPOP expression levels, both in the presence and absence of KFM treatment. Notably, while KFM treatment alone induced significant PD‐L1 downregulation, this effect was markedly attenuated in cells with SPOP knockdown. Quantitative analysis of these results further confirmed these observations (Figure S26, Supporting Information). These findings provide compelling evidence that KFM‐induced PD‐L1 downregulation is likely mediated through enhanced ubiquitination, leading to accelerated proteasomal degradation of PD‐L1. The reversal of KFM's effect on PD‐L1 levels in SPOP‐knockdown cells strongly suggests that SPOP, as part of the CRL3 E3 ligase complex, plays a critical role in this process.

Further supporting the involvement of the ubiquitin‐proteasome pathway, the **Gel@MTX/MET** group exhibited increased expression of UBE2D3, KLHL22, UBE4B, and PSMF1 (Figure S27, Supporting Information), proteins known to be involved in ubiquitination, PD‐1 homeostasis, and proteasome regulation. Additionally, we observed upregulation of the natural killer (NK) cell‐mediated cytotoxicity pathway in the **Gel@MTX/MET** group. This finding aligns with previous reports demonstrating the ability of MET to activate NK cells.^[^ ^33^ ^]^ Taken together, our findings suggest a multifaceted mechanism for the enhanced antitumor immunity observed with **Gel@MTX/MET**, involving both PD‐L1 downregulation via the ubiquitin‐proteasome pathway and augmentation of NK cell activity. These results highlight the potential of this peptide‐based hydrogel system as a novel immunotherapy approach for cancer treatment.

## 3 Conclusion

In summary, our study highlights the efficacy of the peptide **KFM** as a novel regulator of PD‐L1 expression in tumor cells and tissues. We demonstrate that KFM mediates PD‐L1 downregulation through a ubiquitin‐dependent degradation pathway. Furthermore, when self‐assembled into a ROS‐responsive hydrogel (**Gel KFM**), **KFM** synergistically enhances the antitumor effects of MTX and MET. This hydrogel exhibits favorable stability and facilitates controlled drug release, resulting in prolonged cytotoxic effects and significant ICD induction in vitro. Importantly, **Gel KFM** loaded with MTX and MET leads to substantial tumor growth inhibition, downregulation of the immune checkpoint protein PD‐L1, and enhanced CD8+ T cell infiltration in vivo.

Overall, our findings establish the **Gel KFM** delivery platform, in combination with MTX and MET, as a promising chemo‐immunotherapeutic approach. This system effectively targets multiple hallmarks of cancer simultaneously, inhibiting tumor growth, modulating immune checkpoints, and enhancing the efficacy of chemotherapeutic agents. This discovery not only demonstrates that peptides can post‐translationally regulate PD‐L1 stability and cell surface expression, thereby counteracting tumor immune evasion but also provides a proof‐of‐concept that these hydrogel‐forming peptides can actively participate in shaping antitumor immunity, extending beyond their role in drug delivery.

## Conflict of Interest

The authors declare no conflict of interest.

## Supporting information



Supporting Information

## Data Availability

The data that support the findings of this study are available in the supplementary material of this article.

## References

[1] a) F. Voli , E. Valli , L. Lerra , K. Kimpton , F. Saletta , F. M. Giorgi , D. Mercatelli , J. R. C. Rouaen , S. Shen , J. E. Murray , A. Ahmed‐Cox , G. Cirillo , C. Mayoh , P. A. Beavis , M. Haber , J. A. Trapani , M. Kavallaris , O. Vittorio , Cancer Res. 2020, 80, 4129; 32816860 10.1158/0008-5472.CAN-20-0471

[2] J. M. Kim , D. S. Chen , Ann. Oncol. 2016, 27, 1492. 27207108 10.1093/annonc/mdw217

[3] a) M. Yi , X. Zheng , M. Niu , S. Zhu , H. Ge , K. Wu , Mol. Cancer 2022, 21, 28; 35062949 10.1186/s12943-021-01489-2 PMC8780712

[4] P. Wang , Y. Chen , S. Song , T. Wang , W. Ji , S. Li , N. Liu , C. Yan , Front. Pharmacol. 2017, 8, 730. 29093678 10.3389/fphar.2017.00730 PMC5651530

[5] a) A. Naimi , R. N. Mohammed , A. Raji , S. Chupradit , A. V. Yumashev , W. Suksatan , M. N. Shalaby , L. Thangavelu , S. Kamrava , N. Shomali , A. D. Sohrabi , A. Adili , A. Noroozi‐Aghideh , E. Razeghian , Cell Commun. Signal. 2022, 20, 44; 35392976 10.1186/s12964-022-00854-y PMC8991803

[6] a) P. Zhang , Z. Li , W. Cao , J. Tang , Y. Xia , L. Peng , J. Ma , Adv. Mater. 2023, 35, 2305215; 10.1002/adma.202305215 37522451

[7] S. Liang , M. Liu , W. Mu , T. Gao , S. Gao , S. Fu , S. Yuan , J. Liu , Y. Liu , D. Jiang , N. Zhang , Adv. Sci. 2023, 11, 2305275. 10.1002/advs.202305275 PMC10916662 38110834

[8] a) H. Tian , W. Li , G. Wang , Y. Tian , J. Yan , S. Zhou , X. Yu , B. Li , Y. Dai , Adv. Sci. 2023, 10, 2301661; 10.1002/advs.202301661 PMC10375179 37144520

[9] A. Davies , A. Zoubeidi , H. Beltran , L. A. Selth , Cancer Discov. 2023, 13, 1771. 37470668 10.1158/2159-8290.CD-23-0225 PMC10527883

[10] Z. Wang , L. Yuan , X. Liao , X. Guo , J. Chen , J. Med. Chem. 2024, 67, 6027. 38598179 10.1021/acs.jmedchem.3c02143

[11] a) S. Liu , Q. Zhang , H. He , M. Yi , W. Tan , J. Guo , B. Xu , Angew. Chem. Int. Ed. 2022, 61, e202210568; 10.1002/anie.202210568 PMC9869109 36102872

[12] a) Y. Wang , X. Li , D. Zheng , Y. Chen , Z. Zhang , Z. Yang , Adv. Funct. Mater. 2021, 31, 2102505;

[13] a) Y. Liu , Z. Zhou , J. Hou , W. Xiong , H. Kim , J. Chen , C. Zheng , X. Jiang , J. Yoon , J. Shen , Adv. Mater. 2022, 34, 2206121; 10.1002/adma.202206121 36017886

[14] a) X. Liang , Y. Li , B. Guo , Z. Zeng , K. Deng , D. Zou , Y. Xie , Y. Xu , C. Shen , X. Xu , Adv. Funct. Mater. 2024, 34, 2314772;

[15] C. Zhu , L. Ke , X. Ao , Y. Chen , H. Cheng , H. Xin , X. Xu , X. J. Loh , Z. Li , H. Lyu , Q. Wang , D. Zhang , Y. Ping , C. Wu , Y. L. Wu , Adv. Mater. 2023, 36, 2310078. 10.1002/adma.202310078 37947048

[16] a) B. Wu , J. Liang , X. Yang , Y. Fang , N. Kong , D. Chen , H. Wang , J. Am. Chem. Soc. 2024, 146, 8585; 38478659 10.1021/jacs.4c00569

[17] B. Hu , C. Owh , P. L. Chee , W. R. Leow , X. Liu , Y.‐L. Wu , P. Guo , X. J. Loh , X. Chen , Chem. Soc. Rev. 2018, 47, 6917. 29697128 10.1039/c8cs00128f

[18] J. Wang , L. Hu , H. Zhang , Y. Fang , T. Wang , H. Wang , Adv. Mater. 2021, 34, 2302320.

[19] M. Wen , Y. Cao , B. Wu , T. Xiao , R. Cao , Q. Wang , X. Liu , H. Xue , Y. Yu , J. Lin , C. Xu , J. Xu , B. OuYang , Nat. Commun. 2021, 12, 5106. 34429434 10.1038/s41467-021-25416-7 PMC8384847

[20] Y. Xiao , T. Zhang , X. Ma , Q. C. Yang , L. L. Yang , S. C. Yang , M. Liang , Z. Xu , Z. J. Sun , Adv. Sci. 2021, 8, 2101840. 10.1002/advs.202101840 PMC8693073 34705343

[21] E. J. Fox , Neurology 2004, 63, S15. 15623664 10.1212/wnl.63.12_suppl_6.s15

[22] V. Finisguerra , T. Dvorakova , M. Formenti , P. Van Meerbeeck , L. A. Mignion , B. Gallez , B. A. Van den Eynde , J. Böhme , N. Martinez , S. Li , A. Lee , A. Singhal , Nat. Commun. 2020, 11, e005719.

[23] K. Mei , Y. Liao , J. Jiang , M. Chiang , M. Khazaieli , X. Liu , X. Wang , Q. Liu , C. H. Chang , X. Zhang , J. Li , Y. Ji , B. Melano , D. Telesca , T. Xia , H. Meng , A. E. Nel , ACS Nano 2020, 14, 13343. 32940463 10.1021/acsnano.0c05194 PMC8023019

[24] a) J. Cha , W. Yang , W. Xia , Y. Wei , L. Chan , S. Lim , C. Li , T. Kim , S. Chang , H. Lee , J. L. Hsu , H. Wang , C. Kuo , M. Hung , Mol. Cell 2018, 71, 606; 30118680 10.1016/j.molcel.2018.07.030 PMC6786495

[25] a) G. Kim , S. J. Weiss , R. L. Levine , BBA‐Gen. Subjects 2014, 1840, 901; 10.1016/j.bbagen.2013.04.038 PMC3766491 23648414

[26] a) W. Wei , H. Li , G. Zhang , Y. Zhang , K. Wu , R. Bao , G. Wang , H. Zheng , Y. Xia , C. Li , Med. Oncol. 2020, 37, 116; 33215275 10.1007/s12032-020-01445-y

[27] Y. Xiang , L. Chen , C. Liu , X. Yi , L. Li , Y. Huang , Small 2021, 18, 2104591. 10.1002/smll.202104591 34859582

[28] K. Hayashi , F. Nikolos , Y. C. Lee , A. Jain , E. Tsouko , H. Gao , A. Kasabyan , H. E. Leung , A. Osipov , S. Y. Jung , A. V. Kurtova , K. S. Chan , Nat. Commun. 2020, 11, 6299. 33288764 10.1038/s41467-020-19970-9 PMC7721802

[29] D. V. Krysko , A. D. Garg , A. Kaczmarek , O. Krysko , P. Agostinis , P. Vandenabeele , Nat. Rev. Cancer 2012, 12, 860. 23151605 10.1038/nrc3380

[30] X. Xiong , J. Zhao , R. Su , C. Liu , X. Guo , S. Zhou , Nano Today 2021, 39, 101225.

[31] L. Xie , G. Wang , W. Sang , J. Li , Z. Zhang , W. Li , J. Yan , Q. Zhao , Y. Dai , Biomaterials 2021, 269, 120638. 33421711 10.1016/j.biomaterials.2020.120638

[32] M. Zhuang , M. F. Calabrese , J. Liu , M. B. Waddell , A. Nourse , M. Hammel , D. J. Miller , H. Walden , D. M. Duda , S. N. Seyedin , T. Hoggard , J. W. Harper , K. P. White , B. A. Schulman , Mol. Cell 2009, 36, 39. 19818708 10.1016/j.molcel.2009.09.022 PMC2847577

[33] a) M. Foretz , B. Guigas , B. Viollet , Nat. Rev. Endocrinol. 2023, 19, 460; 37130947 10.1038/s41574-023-00833-4 PMC10153049

