# Supplementary material for: Intrinsic PD‐L1 Degradation Induced by a Novel Self‐Assembling Hexapeptide for Enhanced Cancer Immunotherapy
Source: Adv Sci (Weinh). 2024 Nov 12;12(2):2410145. doi: 10.1002/advs.202410145 (PMC11727121; doi:10.1002/advs.202410145)
Supplement: Supplementary file 1 — Supporting Information [file ADVS-12-2410145-s001.pdf]

ADVANCED SCIENCE
Open Access

Supporting Information

for *Adv. Sci.*, DOI 10.1002/advs.202410145

Intrinsic PD-L1 Degradation Induced by a Novel Self-Assembling Hexapeptide for Enhanced Cancer Immunotherapy

Hongxia Zhang, Ming Ji, Yamei Wang, Mengmeng Jiang, Zongyu Lv, Gongyu Li, Lulu Wang* and Zhen Zheng**

<u>**Supplementary Information**</u>

Intrinsic PD-L1 Degradation Induced by a Novel Self-Assembling Hexapeptide for Enhanced Cancer Immunotherapy

Hongxia Zhang#, Ming Ji#, Yamei Wang#, Mengmeng Jiang, Zongyu Lv, Gongyu Li*, Lulu Wang*, Zhen Zheng*

H. Zhang, M. J, M. J, Z. L, Prof. L. Wang, Prof. Z. Zheng
The Province and Ministry Co-sponsored Collaborative Innovation Center for Medical Epigenetics, Tianjin Key Laboratory on Technologies Enabling Development of Clinical Therapeutics and Diagnostics, School of Pharmacy, Tianjin Medical University, Tianjin, 300070, China
E-mail: wanglulu@tmu.edu.cn, zhengzhen90@tmu.edu.cn

Y. Wang, Prof. G. Li
Tianjin Key Laboratory of Biosensing and Molecular Recognition, Research Center for Analytical Science, Frontiers Science Center for New Organic Matter, College of Chemistry, Nankai University, Tianjin 300071, China
E-mail: ligongyu@nankai.edu.cn

TABLE OF CONTENTS

GENERAL

Experimental Section

Supplementary Figures

**Figure S1.**	The MS of hydrogelator **KFM** peptide.
**Figure S2.**	Western blot analysis of PD-L1 expression in MC38 cells with different treatments.
**Figure S3.**	The strain sweep rheological analysis of **Gel KFM** and **Gel KFM** + H_2O_2.
**Figure S4.**	The characterization of **Gel@MTX/MET.**
**Figure S5.**	The MS of hydrogelator **KFM** oxidized product **KFM^o** peptide.
**Figure S6.**	The optical images of **Gel@MTX/MET** before and after incubation with H_2O_2 or PB overnight at 37 °C.
**Figure S7.**	*In vitro* H_2O_2-stimulated disassembly of **Gel@MTX/MET**.
**Figure S8.**	MTT assay of **KFM^o** peptide against 4T1, MC38, and HUVEC cells.
**Figure S9.**	Cell viability of 4T1 and MC38 cells treated with varying concentration of H_2O_2.
**Figure S10.**	Cell apoptosis of MC38 cells after treated with various drug formulations for 24 h.
**Figure S11.**	Quantitative data of the fluorescence intensity of CRT against 4T1 cells.
**Figure S12.**	Fluorescence images of HMGB1 staining in 4T1 cells with treatments.
**Figure S13.**	Quantitative data of the fluorescence intensity of CRT against MC38 cells.
**Figure S14.**	Fluorescence images of HMGB1 staining in MC38 cells with treatments.
**Figure S15.**	The images of MTX and **Gel@MTX** of degradation in BALB/c mice at pre-determined timepoints.
**Figure S16.**	Western blot analysis of PD-L1 expression in 4T1 tumor bearing mice.
**Figure S17.**	Immunofluorescence images showing CD8 and PD-L1 staining in the tumor region of mice at 3 days post-treatment.
**Figure S18.**	Immunofluorescence images showing CD8 and PD-L1 staining in the tumor region of mice at 6 days post-treatment.
**Figure S19.**	Flow cytometric analysis of matured DCs (CD80+CD86+) in tumor tissue of mice after different treatments.
**Figure S20.**	Therapeutic effects of **Gel@MTX/MET** *in vivo*

**Figure S21.**	The enlarged immunofluorescence images of PD-L1 in 4T1 and MC38 tumor bearing mice.
**Figure S22.**	The biocompatibility analysis on 4T1-tumor bearing mice
**Figure S23.**	The biocompatibility analysis on MC38-tumor bearing mice.
**Figure S24.**	GO and KEGG pathway analyses of the differentially expressed proteins.
**Figure S25.**	PCR analysis for amplify and quantify the expression of the PD-L1 mRNA in response to **KFM.**
**Figure S26.**	Quantitative data of the (a) SPOP and (b) PD-L1 expression level of MC38 cells.
**Figure S27.**	Expression levels of selected proteins, with statistical significance indicated.
**Table S1.**	Selected biological processes and pathways for each cluster by hierarchical clustering.

Experimental Section

Reagents and Equipment

All amino acids were purchased from Bide Pharmatech Co., Ltd (Shanghai, China). Rink amide 4-methyl-benzhydrylamine (MBHA) resin and mitoxantrone (MTX) were bought from Bide Pharmatech Co., Ltd (Shanghai, China). Tirapazamine was purchased from HEOWNS Biochemical Technology Co., Ltd (Tianjin, China). Anhydrous N,N-Dimethylformamide was purchased from Meryer Shanghai Chemical Technology Co., Ltd (China). Diisopropylethylamine was bought from Shanghai Macklin Biochemical Co., Ltd (China). O-benzotriazol-1-yl-tetramethyluronium hexafluorophosphate (HBTU) and metformin (MET) were bought from Shanghai Titan Scientific Co., Ltd (China). Peperidine was purchased from Tianjin DaMao Chemical Reagent Factory. Trifluoroacetic acid was bought from Tianjin Kermel Chemical Reagent Co., Ltd. All other organic solvents were purchased from Tianjin Jindong Tianzheng Fine Chemical Reagent Factory (China).

Dulbecco's Modified Eagle Medium (DMEM) and fetal bovine serum (FBS) were purchased from Thermo Fisher scientific (Beijing, China). Phosphate buffered saline (PBS) were purchased from Beijing Solarbio Science&Technology Co., Ltd (China). Penicillin-streptomycin solution, 0.25% Trypsin-EDTA were bought from Beijing Labgic Technology Co., Ltd. 3-(4,5-Dimethylthiazol-2-yl)-2,5-diphenylte-trazolium bromide (MTT) was purchased from HEOWNS Biochemical Technology Co., Ltd. Anti-PD-L1(2B11D11) and anti-GAPDH (60004-1-Ig) were purchased from Proteintech Group, Inc. HRP-labelled Goat anti-Mouse (7076S) was purchased from Cell Signaling Technology. Protease inhibitor (DI101-02) and phosphatase inhibitor (DI201-02) were purchased from Transgen Biotechnology Co., Ltd. SuperSignal West Pico PLUS (34580) was purchased from Thermo Fisher. Lipo8000™ Transfection Reagent (C0533) was purchased from Beyotime Biotechnology.

High performance liquid chromatography (HPLC) analysis was performed on Waters 2695 separations Module. Mass spectra (MS) were analyzed by LTQ-Orbitrap XL mass spectrometer (Thermo scientific, San Jose, CA, USA). Transmission electron

micrograph (TEM) images were obtained on a HT7800 transmission electron microscope (HITACHZ, Japan). Circular dichroism spectra were obtained on J-715 circular dichroism spectrometer (JASCO, Japan). Rheology test was conducted by Discovery HR-20 TA Instruments. UV−vis spectra were obtained on were measured in a microplate reader (Infinite 200 PRO, TECAN) or UV spectrophotometer (U-3900, HITACHI, Japan). Electrophoresis Power Supply, Vertical Electrophoresis System, Protein Transfer Electrophoresis Tank, and Automatic Chemiluminescence Image Analysis System were obtained from Shanghai Tanon Science&Technology Co., Ltd.

Cells and Animals

Mouse breast cancer cells (4T1), mouse colon cancer cells (MC38), and Human Umbilical Vein Endothelial Cells (HUVEC) were gained from the Shanghai Institutes of Biological Science, Chinese Academy of Science. Female BALB/c, C57BL/6J mice (6-8 weeks) were purchased from the SPF Biotechnology Co., Ltd (Beijing, China), and raised under standard conditions. Animal experiments were approved by the Animals Experimentation Ethics Committee of Tianjin Medical University and carried out in accordance with the institutional guidelines (No. TMUaMEC202300).

Peptide Synthesis

The peptides **KFM**, **EFM**, **KFY**, **SFM**, and **RFM** were synthesized by standard Fmoc solid-phase synthesis strategy (SPPS) using Rink amide MBHA resin. The peptide molecular was cleaved from the resin with an admixture of TFA/TIS/H_2O (95:2.5:2.5) for 2 h. The crude peptide was purified using HPLC. The purity was confirmed by HPLC and MS, respectively.

Hydrogel Formation

Gel KFM: The peptide **KFM** (2 mg) was dissolved in phosphate buffer (PB) solution (400 μL, 0.2 M, pH 6.5) and thoroughly dispersed by pipetting. The mixture was then sonicated in a bath sonicator for 1 minute and incubated at 37°C for 6 hours to form the MET/MTX-free hydrogel (**Gel KFM**).

Gel@MTX/MET: Stock solutions of MET and MTX were prepared by dissolving the powders in ultrapure water and DMSO, respectively. The **KFM** peptide was then dispersed in PB solution (0.2 M, pH 6.5) containing MET (40 M) and MTX (250 μM).

This mixture was processed following the same procedure as **Gel KFM** to obtain the MTX/MET-loaded hydrogel (**Gel@MTX/MET**). This method resulted in the near-complete encapsulation of both MTX and MET within the **KFM** hydrogel matrix.

Determination of Encapsulation Efficiency

The drug encapsulation efficiency was determined using UV-Vis spectroscopy. The initial amount of drug added to the hydrogel and the amount remaining within the hydrogel were measured by analyzing the characteristic absorbance at 680 nm for MTX and 233 nm for and MET, respectively. Encapsulation efficiency was calculated using the following formula:[1]

$$\text{Encapsulation Efficiency (\%)} = \left(\frac{\text{Amount of Drug Encapsulated}}{\text{Initial Amount of Drug}}\right) \times 100$$

Rheological Analysis

The rheological properties of **Gel KFM** and **Gel@MTX/MET** were performed using Discovery HR-20 TA rheometer. The frequency sweep measurements were carried out using **Gel KFM** and **Gel@MTX/MET** (0.5%, w/w). The storage modulus (G′) and loss modulus (G′′) were detected keeping strain at 0.1% with a continuous frequency (0.1-10 Hz). To examine the thixotropic property of **Gel KFM** and **Gel@MTX/MET**, the dynamic strain sweep (0.01-10%) at constant 1 rad/s of frequency were inspected, followed the storage modulus (G′) and loss modulus (G′′) were measured.

Circular Dichroism (CD) Spectra

The circular dichroism spectra of **Gel KFM** (1 mM) and **Gel@MTX/MET** (1 mM) were detected at 25 °C using J-715 circular dichroism spectrometer with a 2 mm path length quartz cuvette. All spectra (190-280 nm) were recorded at 1 nm intervals in PB solution.

Transmission Electron Microscopy (TEM)

The self-assembled peptides **KFM**, **EFM**, **KFY**, **SFM**, and **RFM**; **Gel KFM** (1 mM) and **Gel@MTX/MET** (1 mM) were deposited onto a copper grid, which were incubated 5 min, and then the hydrogel was dried by bibulous paper. Subsequently, the samples were air-dried overnight. The TEM images were captured with a transmission

electron microscope.

ROS-Responsiveness Evaluation of the Hydrogel

The ROS-responsiveness of the hydrogel **Gel KFM** was verified by HPLC. The **Gel KFM** or **Gel@MTX/MET** was cultured with H_2O_2 at 37 °C in PB solution overnight, and then the resulting reaction solution was subjected to HPLC analysis.

***In Vitro* Drug Release**

The 300 μL of hydrogel **Gel KFM** and **Gel@MTX/MET** were placed on the bottom of 2.0 mL centrifuge tube, and the PB buffer (300 μL) with or without H_2O_2 (100 μM) was added on the top of hydrogel layer at 37 °C. Whereafter, at the indicated time points, the released solution was collected, and equal volume of fresh PB buffer was then added to the tube. The collected top layer solution was detected using microplate reader or UV spectrophotometer.

***In Vitro* Cell Cytotoxicity and Cell Apoptosis Assay**

The cytotoxicity of **KFM** and **KFM^o** were assessed as follows. Briefly, 4T1, MC38 and HUVEC cells were seeded on 96-well plate (5000 cells per well) overnight. The cells were incubated with 100 μL fresh DMEM containing with various concentration of for 24 h, respectively. Moreover, the 4T1 and MC38 tumor cells co-cultured with DMEM containing various concentration of MTX for 24 h. And then, the cells viability was evaluated using the MTT assay.

For the cytotoxicity of **Gel@MTX** and **Gel@MTX** with H_2O_2, 10 μL hydrogel **Gel@MTX** were placed on the bottom of 96-well plate and cured overnight. Subsequently, the cells at a density of 1×10^4 were cultivated into 96-well plate and then cultured with or without H_2O_2 (5 μM or 100 μM) for 24 h. The cells viability was evaluated using the MTT assay.

To assess cell apoptosis induced by **Gel@MTX**, 4T1 cells were seeded in 6-well plates at a density of 2×10^5 cells/well. Cells were then treated with PBS, free MTX (5 μM), or **Gel@MTX** for 24 hours. Following incubation, all cells were harvested and stained with Annexin V and propidium iodide (PI) according to the manufacturer's protocol. Apoptosis was immediately analyzed using flow cytometry.

***In vitro* Immunogenic Cell Death Assay**

For the induction of immunogenic cell death by chemotherapeutic agent MTX, the presence of DAMPs within indicated treatments were examined by calreticulin (CRT) and high mobility group box 1 protein (HMGB1) expression level. Firstly, 5×10^4 tumor cells were seeded in 24-well plate, and culture with PBS, free MTX, and **Gel@MTX** for 12 h. And then, fixed with 4% formaldehyde for 30 min at room temperature, and washed twice with PBS. A blocking solution of 10% goat serum was added to the wells and incubated for 50 min to minimize nonspecific antibody adsorption. The cells were treated with primary antibodies overnight at 4 °C, subsequently, stained with FITC-labeled secondary antibodies for 2 h, followed by mounting solution with DAPI, images captured by fluorescence microscopy (Leica DMI3000B). The fluorescence intensity was analyzed using Image J.

Western Blotting Analysis

Cellular proteins were extracted with the lysis buffer containing 8 M urea, 0.05 M Tris-HCl (pH 7.5), 1% β-Mercaptoethanol, 1% protease inhibitor, and 1% phosphatase inhibitor, while tumor protein was extracted with the medium RIPA lysis buffer containing 1% protease inhibitor and 1% phosphatase inhibitors by 1-minute ultrasonication, then kept on ice for 30 minutes and microcentrifugated at 12,000 g for 15 minutes at 4 °C to remove debris. Immunoblotting was performed using 30 μg and 50 μg of protein lysates from cells and tumors, respectively. All samples were resolved in SDS-PAGE, transferred to polyvinylidene difluoride membranes and blocked with 5% non-fat milk. After blocking, the PVDF membranes were incubated with primary antibodies: PD-L1 (1:2000) and GAPDH (1:20000) and then incubated with the secondary antibody HRP-labelled Goat anti-Mouse (1:2000). After washing with PBST buffer three times, PVDF membranes were followed by signal detection using ECL reagents. GAPDH is used as a loading control.

RNA Interference

Spop knockdown was achieved using siRNA. Transfection was performed with Lipo8000™ Transfection Reagent according to the manufacturer's instructions. The siRNA sequences were:

Spop siRNA-1:

Sense: 5'-GGUGCUAUACACAGAUCAATT-3'

Antisense: 5'-UUGAUCUGUGUAUAGCACCTT-3'

Spop siRNA-2:

Sense: 5'-GGCUCACAAGGCUAUCUUATT-3'

Antisense: 5'-UAAGAUAGCCUUGUGAGCCTT-3'

In Vivo **Therapeutic Efficacy and Immune Activation**

Animal experiments were conducted in compliance with the institutional guidelines and approved by the Animals Experimentation Ethics Committee of Tianjin Medical University (No. TMUaMEC2023008). BALB/c mice were injected subcutaneously with 100 μL of either MTX or **Gel@MTX**. To assess gel retention and degradation over time, mice were euthanized at 12, 36, 60, and 72 hours post-injection, and the injection sites were imaged.

The 4T1 tumor-bearing mice with an average tumor volume of 60 mm^3 were randomly dispersed into 4 groups (n = 5) as follows: PBS, MTX (3.0 mg/kg), **Gel@MTX**, and **Gel@MTX/MET**. The hydrogel formulations and free MTX were peritumoral injected at an equivalent dose of MTX (3.0 mg/kg) or MET (139 mg/kg). The treatments were implemented on day 0 and 3. The mice tumor volume and body weight were observed every two days during treatment period. Tumor volume was calculated by the following equation: volume = length × $(\text{width})^2/2$. On days 3, 6, and 18, mice were euthanized. Major organs and tumor tissues were harvested for immunohistochemistry staining. Additionally, tumor and spleen tissues were collected for flow cytometry analysis.

At day 18, all mice were euthanized and the major organs along with tumor tissues were dissected and sent for immunohistochemistry staining. The tumor and spleen were also harvested and analyzed by flow cytometry.

The MC38 tumor-bearing mice with an average tumor volume of 70 mm^3 were randomly dispersed into 5 groups (n = 5) as follows: PBS, MTX (1.5 mg/kg), **Gel@MTX**, MET/MTX, and **Gel@MTX/MET**. The hydrogel formulations and free MTX were peritumoral injected at an equivalent dose of MTX (1.5 mg/kg) or MET

(69.5 mg/kg). The treatments were implemented on day 0 and 3. Finally, the measurements were performed as described above.

Flow cytometry analysis

T-lymphocytes: Cells were stained with FITC-conjugated anti-CD3, APC-conjugated anti-CD4, and PE-conjugated anti-CD8 antibodies for 30 minutes in the dark at 4°C. CD3+ cells were gated for analysis of CD4+ and CD8+ T cell populations. Data were analyzed using FlowJo software (FlowJo, LLC).

Dendritic cells: Cells were stained with FITC-conjugated anti-CD45, V450-conjugated anti-CD80, and APC-conjugated anti-CD86 antibodies for 30 minutes in the dark at 4°C. CD45+ cells were gated for analysis of CD80+ and CD86+ dendritic cell populations. Data were analyzed using FlowJo software (FlowJo, LLC).

Data statistical analysis

The statistical data were analyzed using GraphPad Prism v6.0 software and Origin 8.0 software. All data were shown as the mean ± standard deviation (SD). Statistical comparisons were assessed using unpaired t-test for p values (*p <0.05, **p <0.01, ***p <0.001, ****p <0.0001).

Proteomics analysis

For pairwise comparisons between groups, we conducted Student's t-tests and visualized the results using volcano plots. Colored dots represent significantly differentially expressed proteins (DEPs) identified with a p-value < 0.05 and fold change > 1.5. Analysis of DEPs provided insights into protein abundance variations across treatments. Hierarchical clustering of DEPs revealed distinct treatment differences, forming four clusters.

Supplementary Figures

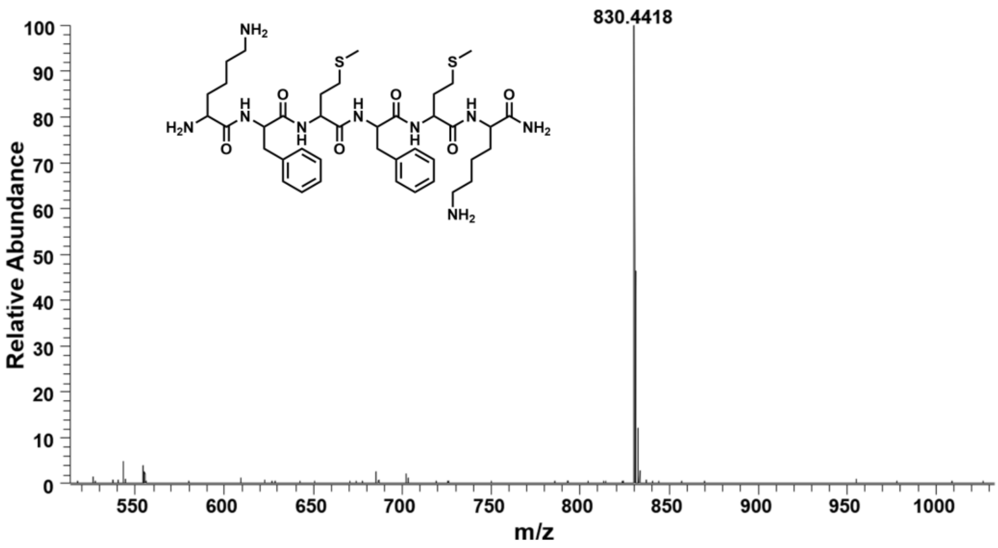

Figure S1. MS spectrum of **KFM**. MS calculated for **KFM** $[(M+H)^+]$: 830.4421; obsvd. $[(M+H)^+]$: m/z 830.4418.

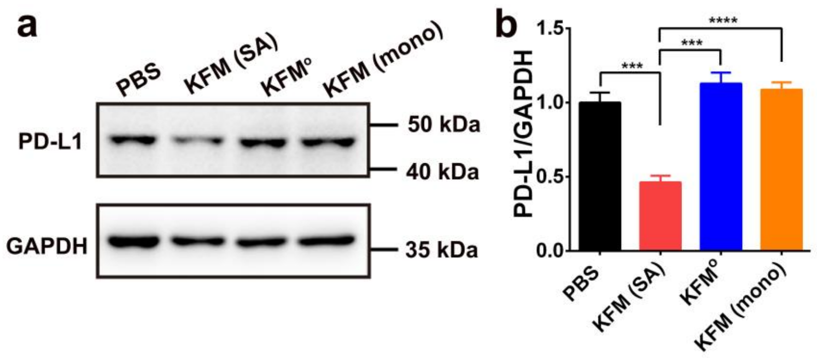

Figure S2. Western blot analysis of PD-L1 expression in MC38 cells following treatment with self-assembled **KFM** (SA), **KFM°**, and monomeric **KFM** (mono). b) Quantification of PD-L1 expression levels. ***P < 0.001. ****P < 0.0001.

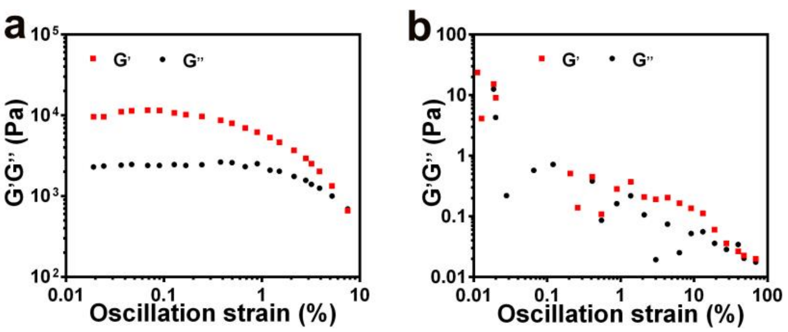

Figure S3. The strain sweep (0.01-100%) rheological analysis of the (a) **Gel KFM** and (b) **Gel KFM** hydrogel after incubation with 10 mM H_2O_2 overnight (frequency: 1 rad/s).

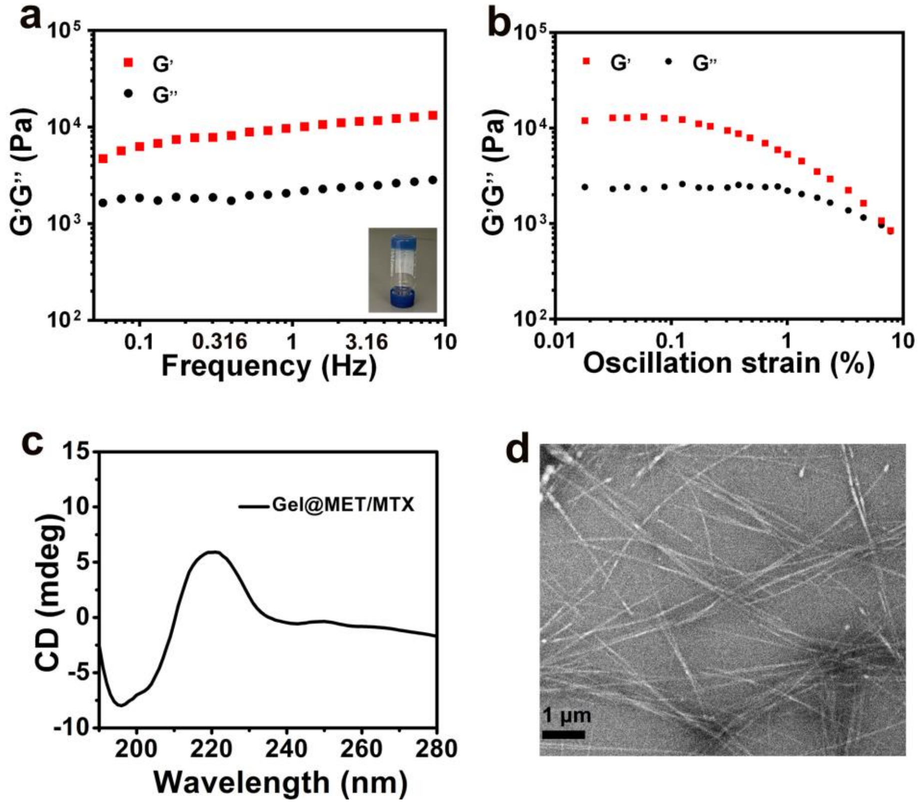

Figure S4. Characterizations of **Gel@MTX/MET** hydrogel. (a) The frequency sweep rheological analysis of the **Gel@MTX/MET** (Insets: images of corresponding hydrogels). (b) The strain sweep rheological analysis of **Gel@MTX/MET** hydrogel. (c) The Circular dichroism (CD) spectrum of **Gel@MTX/MET** hydrogel. (d) The Representative TEM images of **Gel@MTX/MET**.

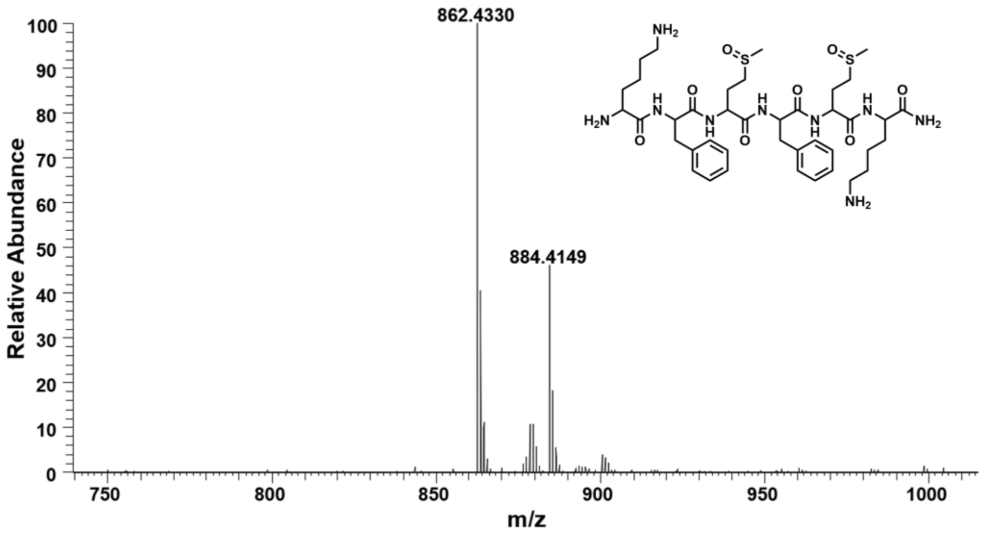

Figure S5. MS spectrum of **KFM°**. MS calculated for **KFM°** [$(M+H)^+$] : 862.4421; [$(M+Na)^+$] : 884.4139; obsvd. [$(M+H)^+$]: m/z 862.4330; [$(M+Na)^+$]: m/z 884.4149.

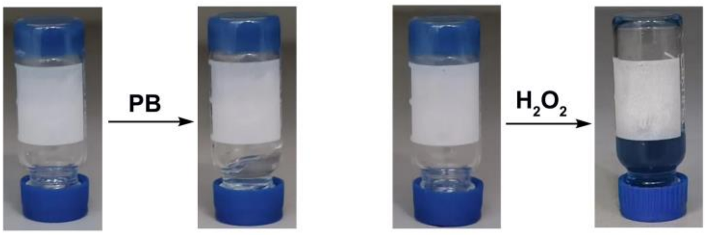

Figure S6. Optical images of 0.5 wt% **Gel@MTX/MET** before and after incubation with H_2O_2 (10 mM) or PB (pH 6.5) overnight at 37 °C, respectively.

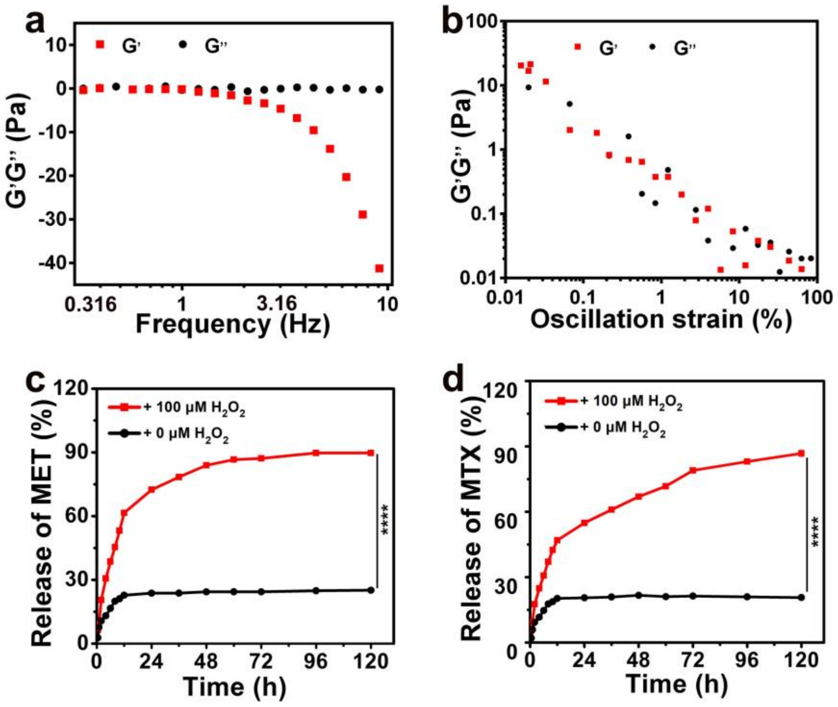

Figure S7. *In vitro* H_2O_2-stimulated disassembly of **Gel@MTX/MET**. (a) The frequency sweep (0.1-10 Hz) rheological analysis of 0.5 wt% **Gel@MTX/MET** after incubation with 10 mM H_2O_2 overnight (strain: 0.1%). (c) MET and (d) MTX in culture medium after incubation **Gel@MTX/MET** with or without 100 μM H_2O_2 at predetermined time points, respectively. ****p <0.0001.

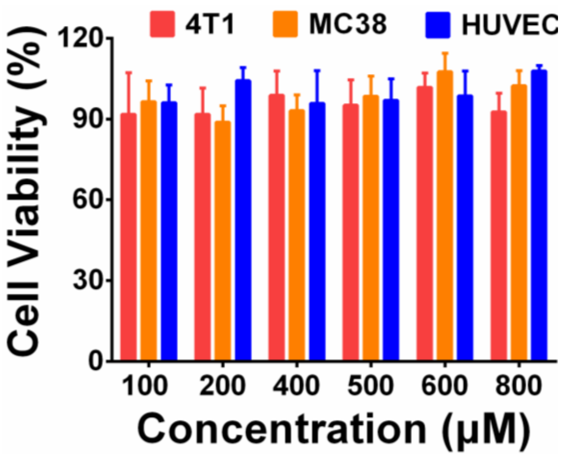

Figure S8. Cell viability of 4T1, MC38, and HUVEC cells treated with **KFM[o]** peptide, as determined by MTT assay.

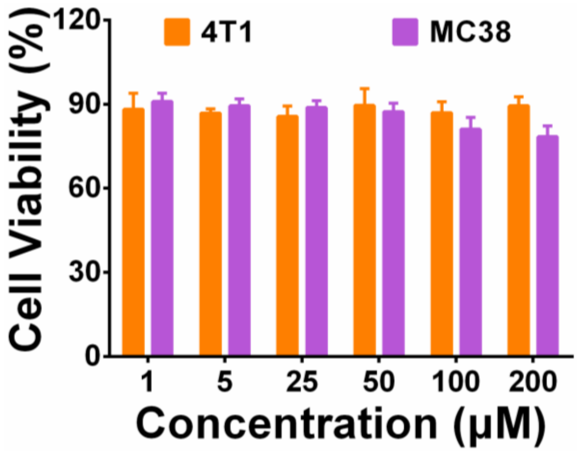

Figure S9. Cell viability of 4T1 and MC38 cells treated with varying concentration of H_2O_2.

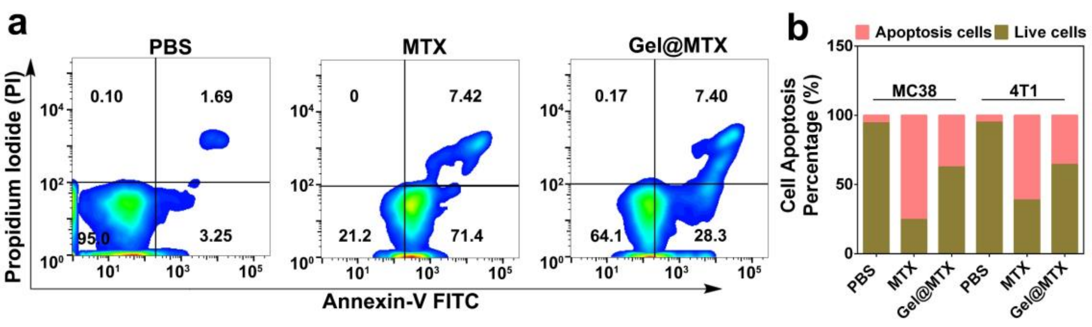

Figure S10. Apoptosis analysis of MC38 cells after 24-hour treatment with various drug formulations. (a) Flow cytometry plots showing cell apoptosis. (b) Quantitative analysis of apoptotic cells based on flow cytometry data.

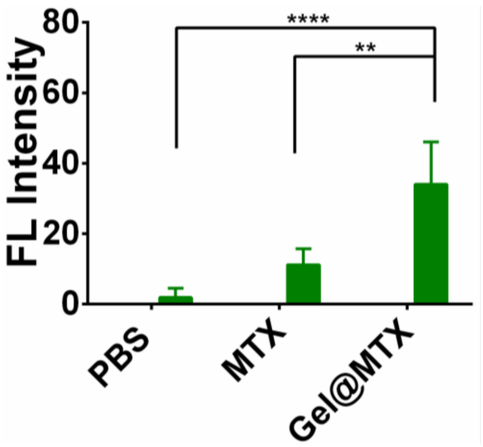

Figure S11. Quantitative data of the fluorescence intensity of CRT against 4T1 cells. $^{**}P < 0.01$, $^{****}P < 0.0001$.

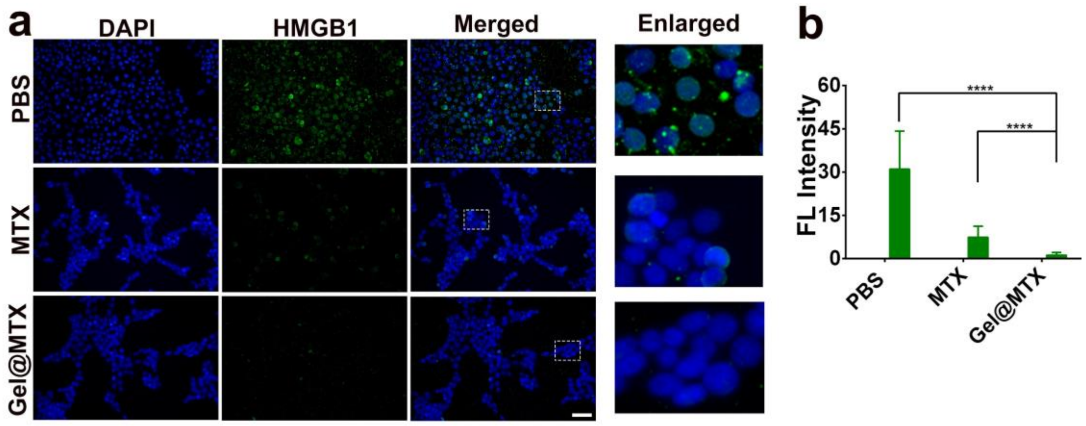

Figure S12. (a) The fluorescence images of HMGB1 in 4T1 cells after treatment with PBS, MTX, and **Gel@MTX**. Scale bar: 100 μm. (b) Quantitative data of the fluorescence intensity in (a). $^{****}P < 0.0001$.

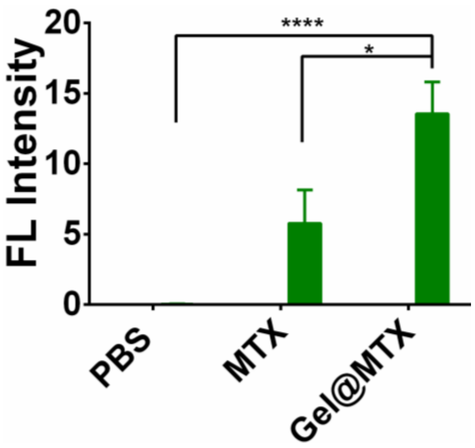

Figure S13. Quantitative data of the fluorescence intensity of CRT against MC38 cells. $^{*}P < 0.05$, $^{***}P < 0.0001$.

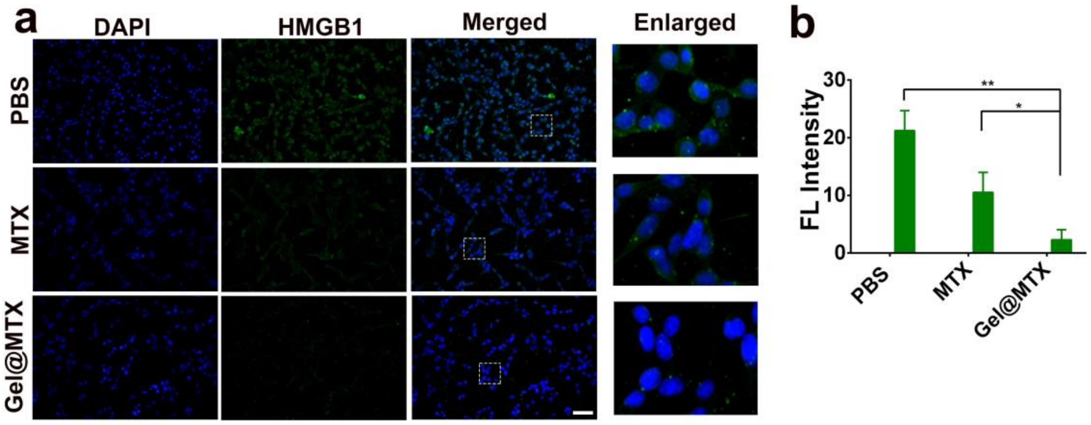

Figure S14. (a) The fluorescence images of HMGB1 in MC38 cells after treatment with PBS, MTX, and **Gel@MTX**. Scale bar: 100 μm. (b) Quantitative data of the fluorescence intensity in (a). *$P <$ 0.05, **$P < 0.01$.

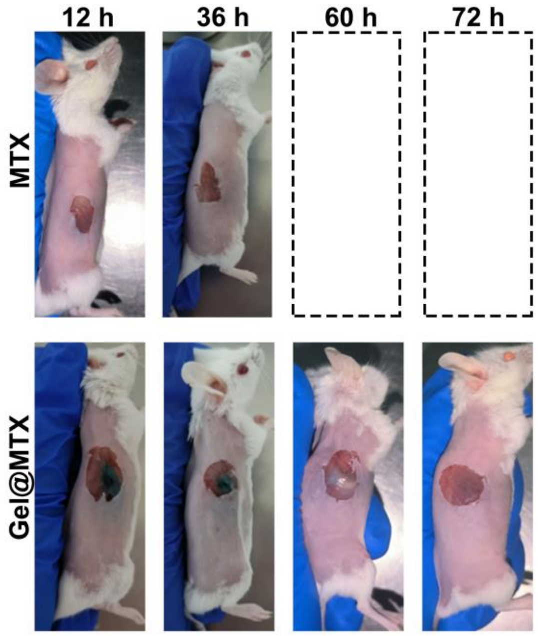

Figure S15. *In vivo* degradation of MTX and **Gel@MTX** in BALB/c mice. (Images show the injection sites at 12, 36, 60, and 72 hours post-administration).

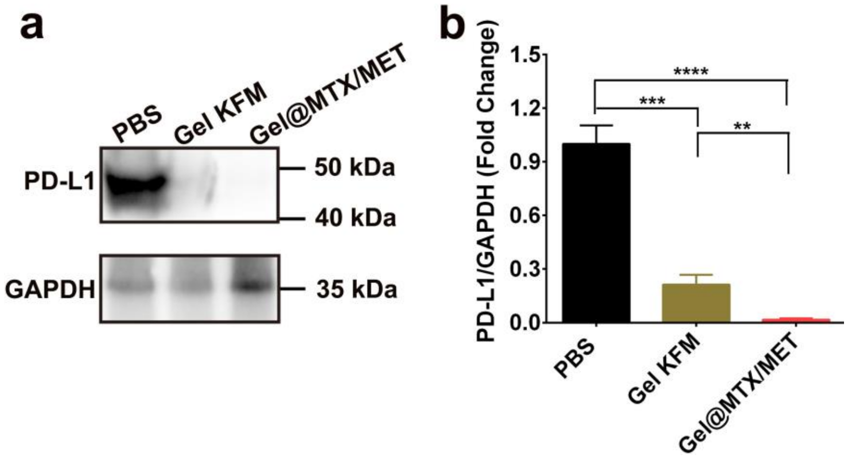

Figure S16. (a) Western blot analysis of PD-L1 expression in 4T1 bearing mice after treatment with **Gel KFM** and **Gel@MTX/MET**. (b) Quantitative data of PD-L1 expression levels in 4T1 tumor bearing mice. ***p* <0.01, ****p* <0.001, *****p* <0.0001.

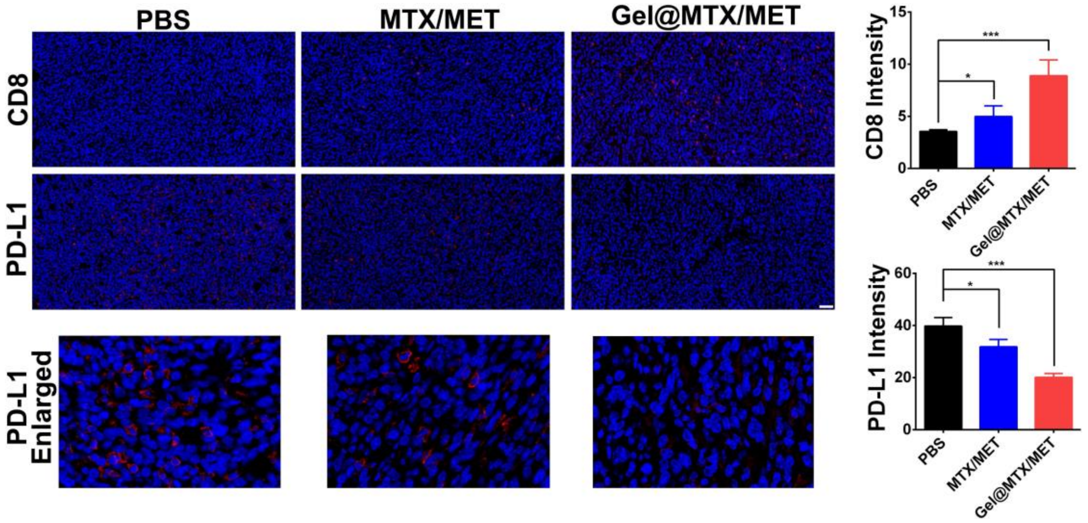

Figure S17. CD8 and PD-L1 expression in 4T1 tumor tissues 3 days post-treatment. Left: Immunofluorescence images of CD8 and PD-L1 staining in tumor sections. Right: Quantification of CD8 and PD-L1 fluorescence intensity. Scale bar: 50 µm. **P* < 0.05, ****P* < 0.001.

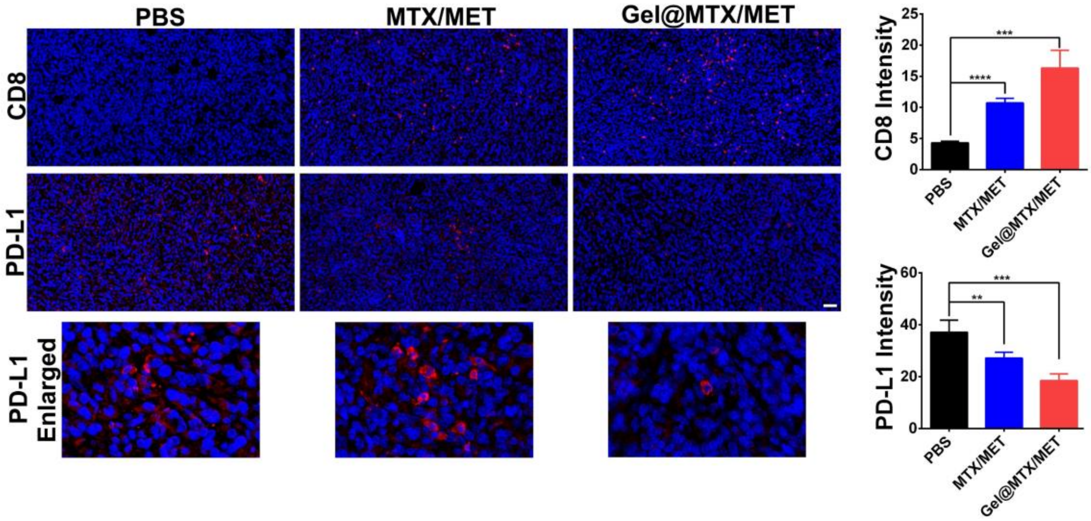

Figure S18. CD8 and PD-L1 expression in 4T1 tumor tissues 6 days post-treatment. Left: Immunofluorescence images of CD8 and PD-L1 staining in tumor sections. Right: Quantification of CD8 and PD-L1 fluorescence intensity. Scale bar: 50 μm. *$P < 0.05$, ***$P < 0.001$.

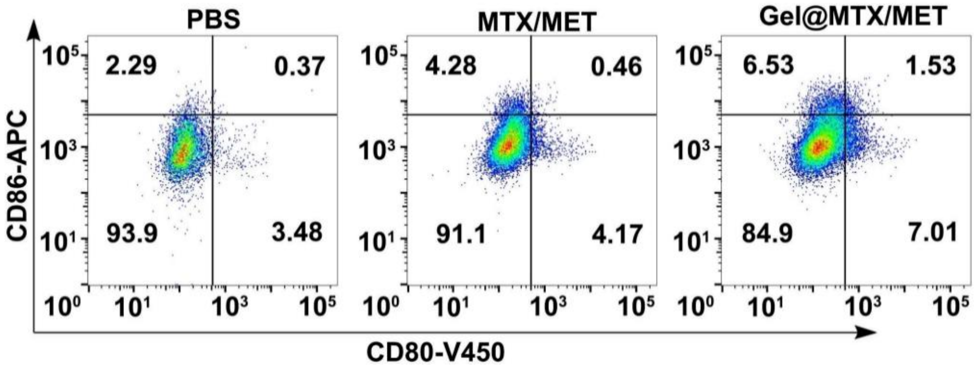

Figure S19. Flow cytometric analysis of matured DCs (CD80+CD86+) in 4T1 tumor tissue of mice after different treatments.

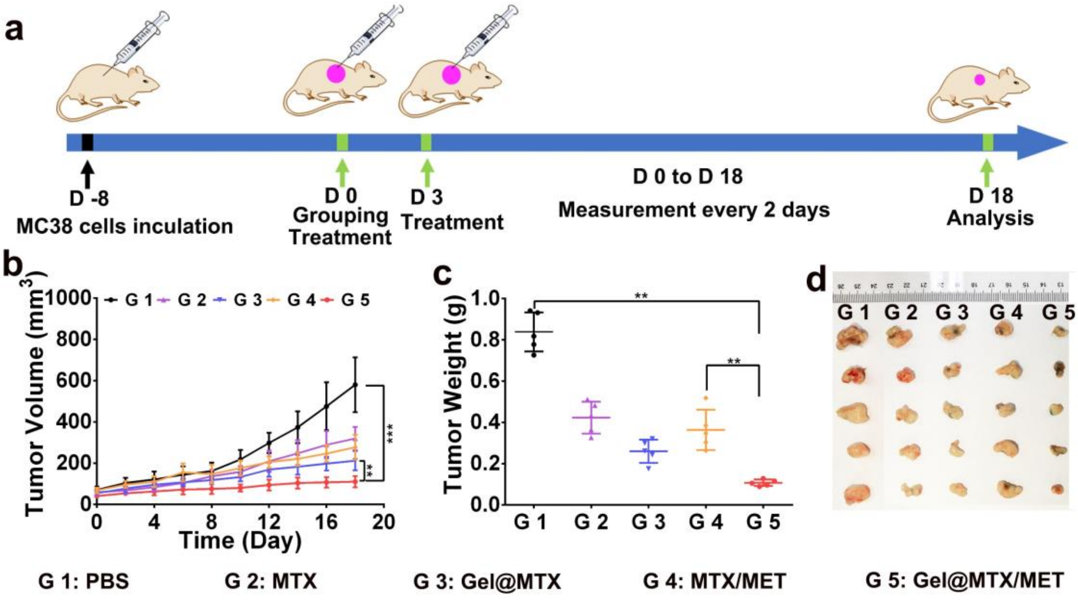

Figure S20. Therapeutic effects of **Gel@MTX/MET** *in vivo*. (a) Schematic iconography of therapeutic protocol of the mice bearing MC38 tumors. (b) Tumors growth curve for mice intratumorally injected with PBS, MTX, **Gel@MTX**, MTX/MET, and **Gel@MTX/MET**. (c) Tumors weight of 4T1-tumor bearing mice after sacrifice. (d) Representative tumors images of all the mice at 18 days post-treatment. G1: PBS, G2: MTX, G3: **Gel@MTX**, G4: MTX/MET, G5: **Gel@MTX/MET**.

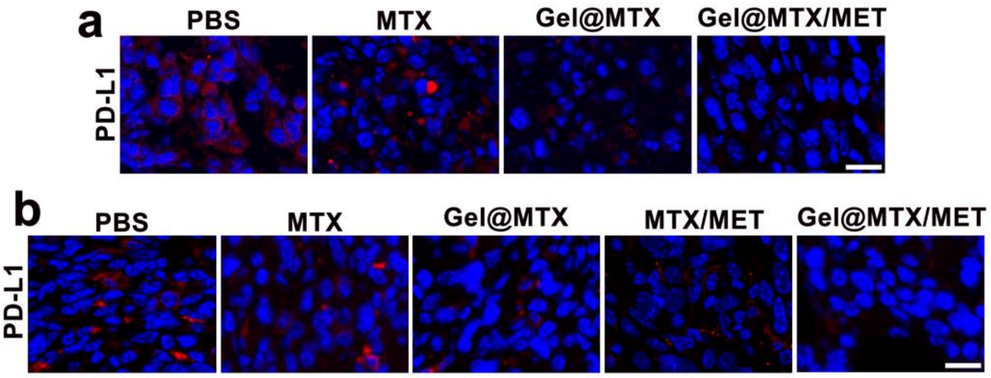

Figure S21. The enlarged immunofluorescence images of PD-L1 in (a) 4T1 tumor and (b) MC38 tumor bearing mice. Scale bar: 20 μm.

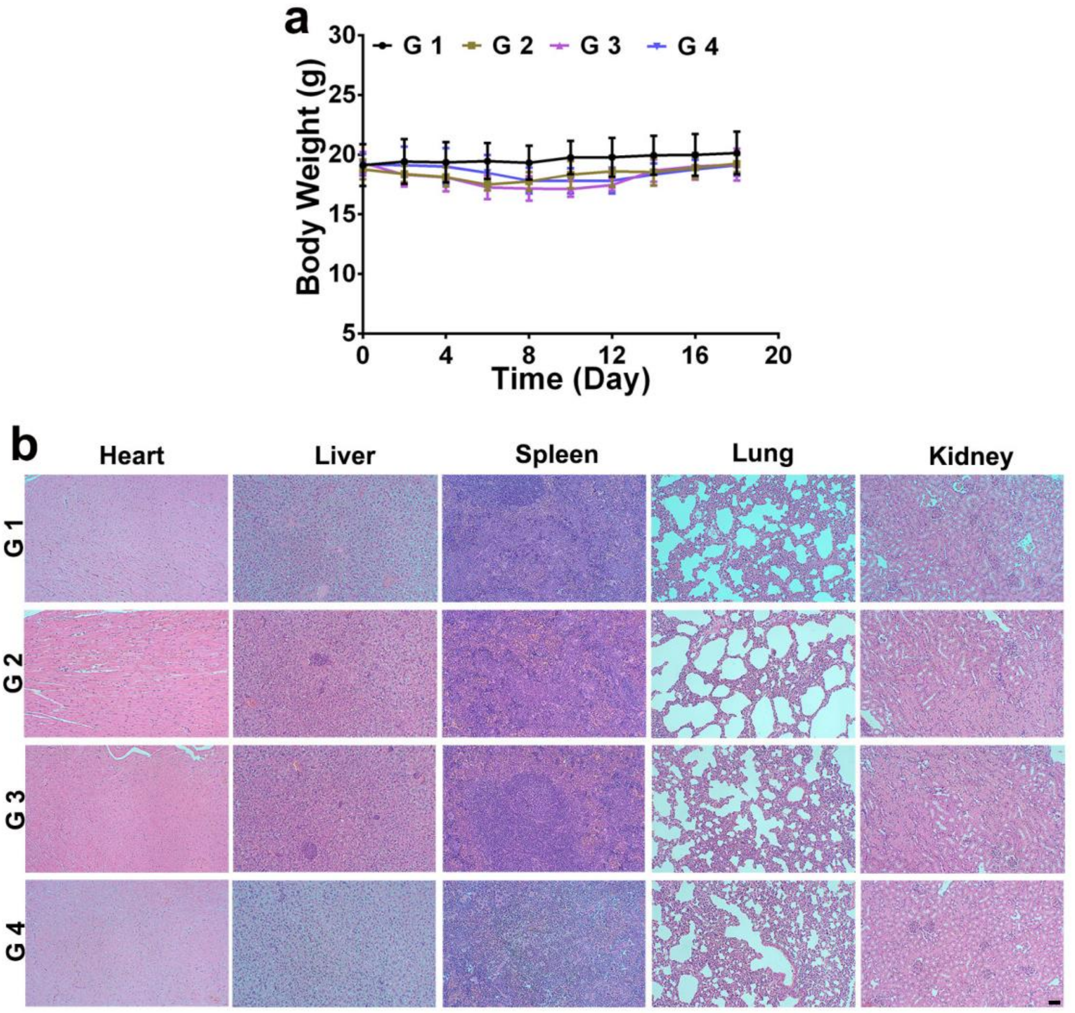

Figure S22. The biocompatibility analysis on 4T1-tumor bearing mice. (a) Body weight change of the mice in different treatments (n = 5). (b) Representative photographs of H&E-stained major organs from mice with different treatments (G 1: PBS, G 2: MTX, G 3: **Gel@MTX**, G 4: **Gel@MTX/MET**). Scale bar: 50 μm.

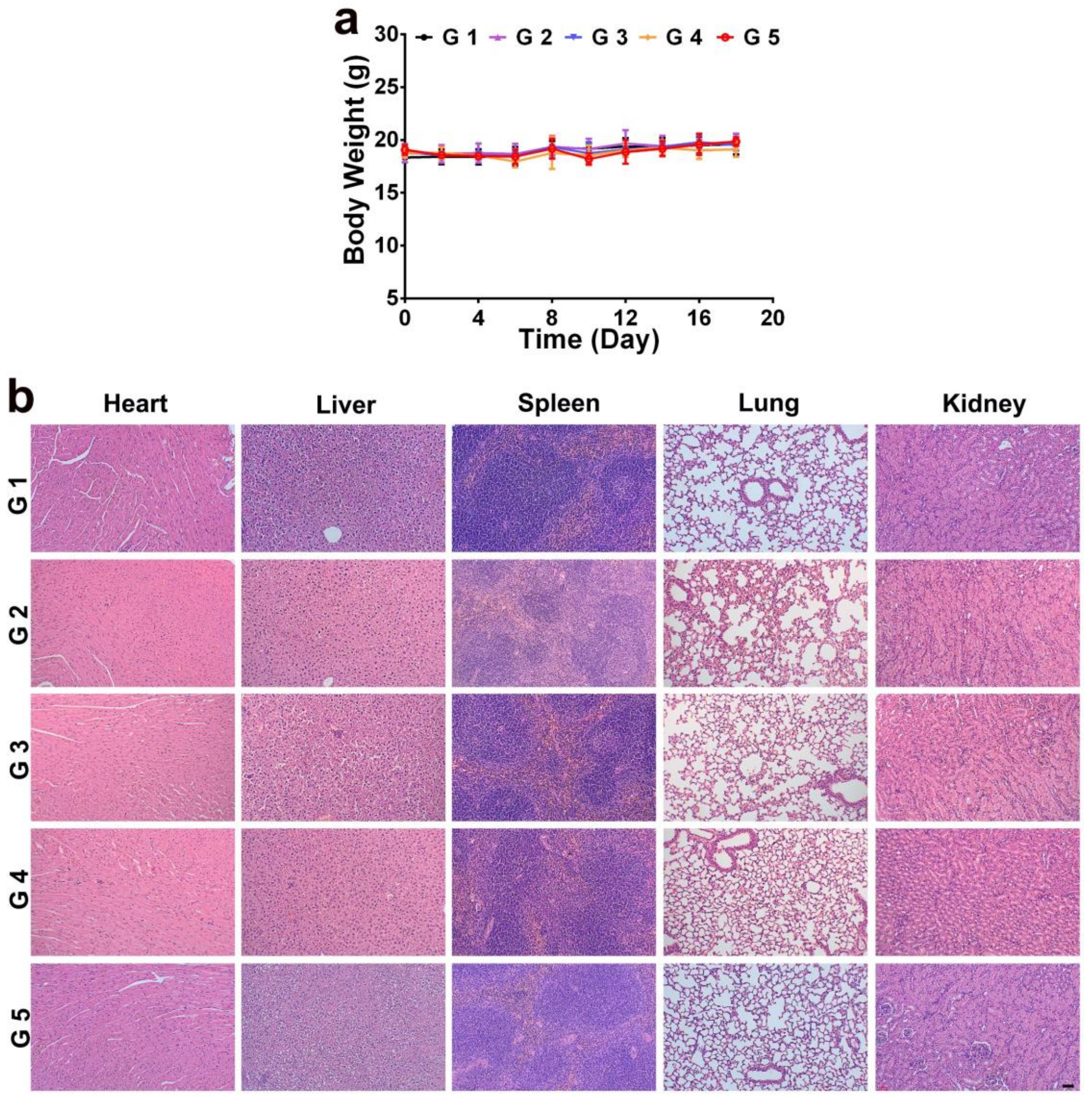

Figure S23. The biocompatibility analysis on MC38-tumor bearing mice. (a) Body weight change of the mice in different treatments (n = 5). (b) Representative photographs of H&E-stained major organs from mice with different treatments (G 1: PBS, G 2: MTX, G 3: **Gel@MTX**, G 4: MTX/MET, G 5: **Gel@MTX/MET**). Scale bar: 50 μm.

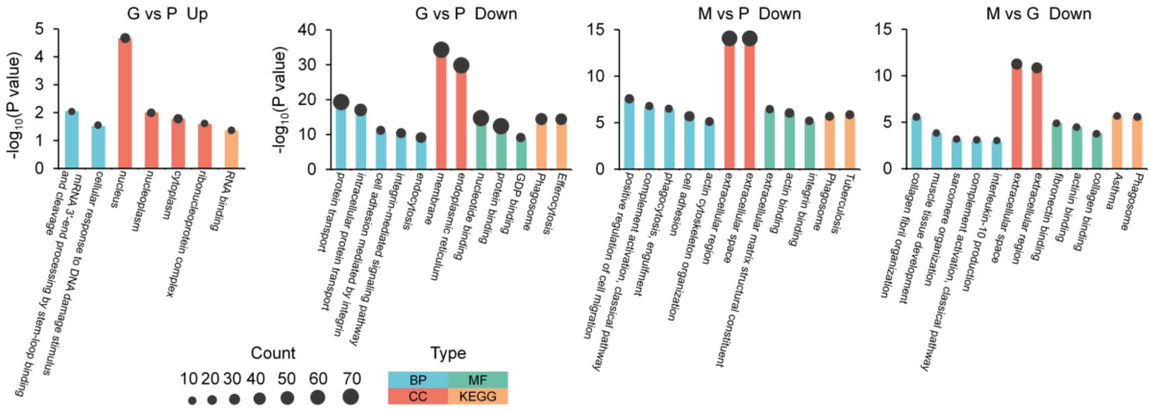

Figure S24. GO and KEGG pathway analyses of the differentially expressed proteins. PBS (P), **Gel KFM** (G), and **Gel@MTX/MET** (M).

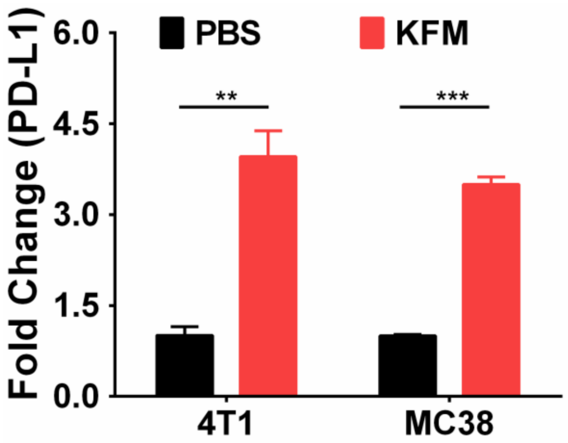

Figure S25. PCR analysis for amplify and quantify the expression of the PD-L1 mRNA in response to **KFM**. $**p < 0.01$, $***p < 0.001$.

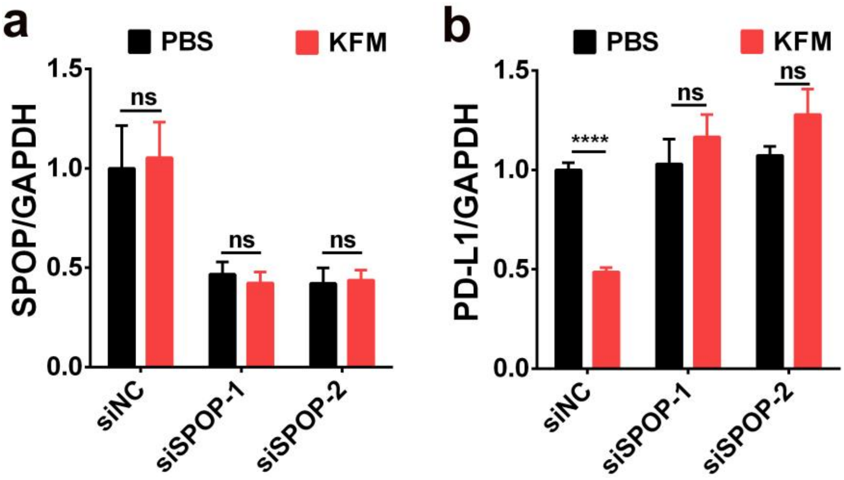

Figure S26. Quantitative data of the (a) SPOP and (b) PD-L1 expression level of MC38 cells. $****P < 0.0001$.

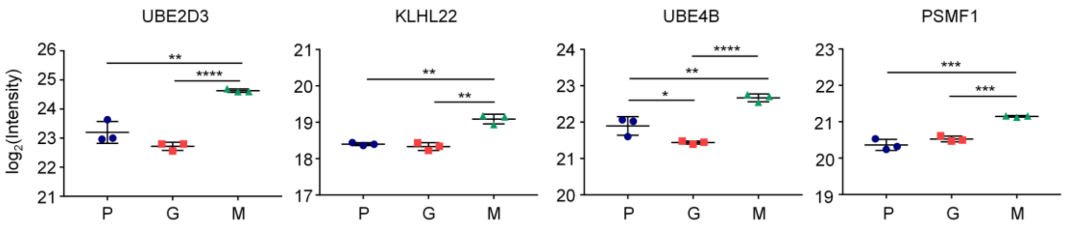

Figure S27. Expression levels of selected proteins, with statistical significance indicated. (*$p < 0.05$, **$p < 0.01$, ***$p < 0.001$, ****$p < 0.0001$). PBS (P), **Gel KFM** (G), and **Gel@MTX/MET** (M).

Table S1. Selected biological processes and pathways for each cluster by hierarchical clustering.

Cluster	Term	Count	P value
Cluster 1	immune system process	32	3.28E-05
Cluster 1	innate immune response	30	6.52E-05
Cluster 1	antigen processing and presentation	8	7.97E-05
Cluster 1	regulation of innate immune response	6	1.87E-04
Cluster 2	ubiquitin-dependent protein catabolic process	31	6.65E-05
Cluster 2	positive regulation of apoptotic process	41	7.74E-04
Cluster 2	protein K11-linked ubiquitination	7	0.008084
Cluster 2	regulation of proteasomal ubiquitin-dependent protein catabolic process	5	0.010508
Cluster 3	collagen fibril organization	10	5.19E-08
Cluster 3	complement activation, classical pathway	9	2.28E-07
Cluster 3	actin cytoskeleton organization	15	2.22E-06
Cluster 3	actin filament organization	12	6.21E-06
Cluster 4	rRNA processing	47	2.49E-28
Cluster 4	ribosome biogenesis	35	8.17E-21
Cluster 4	methylation	19	2.78E-04
Cluster 4	regulation of apoptotic process	18	0.006457

Reference

[1] B. Farasati Far, M. Safaei, R. Nahavandi, A. Gholami, M. R. Naimi-Jamal, S. Tamang, J. E. Ahn, M. Ramezani Farani, Y. S. Huh, *ACS Omega* **2024**, 9, 29139.
